# Recent advances in the use of benzimidazoles as corrosion inhibitors

**DOI:** 10.1186/s13065-019-0655-y

**Published:** 2019-12-23

**Authors:** Maria Marinescu

**Affiliations:** Department of Organic Chemistry, Biochemistry and Catalysis, Faculty of Chemistry, University of Bucharest, 90-92 Şoseaua Panduri, sector 5, 050663 Bucharest, Romania

**Keywords:** Corrosion science, Organic synthesis, Nanomaterials, Electrochemical impedance spectroscopy, Inhibition efficiency

## Abstract

**Background:**

Benzimidazole, a key heterocycle in therapeutic chemistry, and its derivatives, are recently mentioned in the literature as corrosion inhibitors for steels (CS, MS), pure metals (Fe, Al, Cu, Zn) and alloys. Benzimidazoles are good corrosion inhibitors for extremely aggressive, corrosive acidic media such as 1 M HCl, 1 M HNO_3_, 1.5 M H_2_SO_4_, basic media, 0.1 M NaOH or salt solutions. Benzimidazole derivatives act as mixed type inhibitors, exhibiting stronger inhibitive effect on the cathodic reaction than on the anodic one.

**Conclusion:**

These review highlights recent research in the field of benzimidazole compounds that their role as corrosion inhibitors, the structure of the compounds, electrochemical studies, the experimental conditions, the proposed mechanisms as well as the quantum theoretical studies that predict the structure of the compounds with inhibition properties.
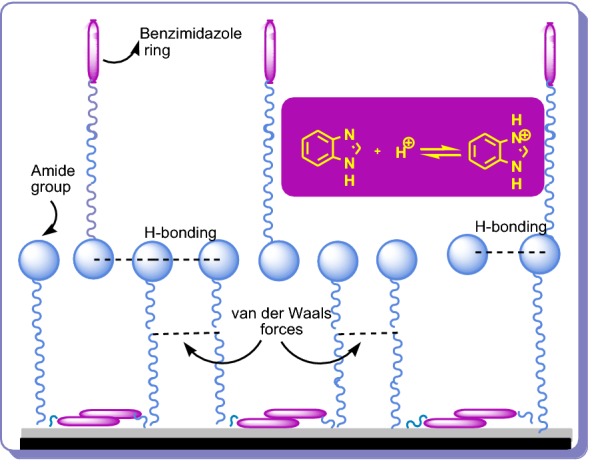

## Background

Corrosion is a serious problem, of great relevance in a wide range of industrial applications and products [1, 2]. Despite breakthrough in the field of corrosion science and technology, the unwanted corrosion process remains a major obstacle for industries all over the world. Corrosion of materials is one of the main problems in industry that is associated to significant economic losses, such as: loss or contamination of the product, reduction in efficiency, increase of maintenance needs, plant shutdowns and expensive overdesign [2]. Upgrading materials, process control, chemical inhibition and blending of production fluids are different ways for preventing corrosion damage. Corrosion inhibitors are synthetic or natural substances which, added in small amounts to a corrosive solution, decrease the rate of attack by the environment on metals [3, 4].

Several authors reported the effectiveness of organic inhibitors which generally protect the metal from corrosion by forming a film on the metal surface [4]. There are many classes of organic and inorganic compounds investigated for their properties of protection of metal in different corrosive media. Organic compounds with multiple bonds, compounds which contain heteroatoms (such as N, P, S, O) or those possessing certain functional groups such as –NH_2_, –COOH or –OH (amines, acids, alcohols, phenols, aminoacids, etc.) are mentioned in literature to be effective corrosion inhibitors [3].

Although organic compounds preferred to be corrosion inhibitors are “green”, such as amino acids, drugs, surfactants, biopolymers, or ionic liquids, there is a pronounced preference for heterocyclic organic compounds, especially those containing nitrogen, sulfur or oxygen, such as imidazoles, triazoles, thiazoles, benzimidazoles, benzotriazoles, purine or adenine [5].

**Fig. 1:**
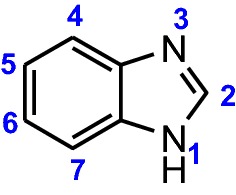
Chemical structure of benzimidazole **1**

Benzimidazole **1** (Fig. 1), a heterocyclic aromatic compound discovered by Hoebrecker, part of vitamin B_12_, is one of the most prominent heterocycles, very known for the therapeutic properties of its compounds [6, 7], but very used in many others fields, like electronics, dyes, pigments, aeronautic industry, optical sensing, catalysts, electrolyte for fuel cells, fungicides, herbicides, etc. [8–11]. Recent literature reported benzimidazole and its derivatives as corrosion inhibitors (CIs) in relation to spatial molecular structure, surface charge density, the electronic parameters and their affinity for the metal surface [2, 12]. Also, the subject of CIs, becomes very important if we mention that the petroleum industry is the largest consumer of CIs. Benzimidazoles possess appropriate structures to coordinate metals; therefore they have ability to control the corrosion of steel or metals. Several papers propose various mechanisms of action in which benzimidazoles are adsorbed on the metal surface [2, 13].

In this paper we propose to present most of the benzimidazole derivatives which are mentioned in literature for their properties of corrosion inhibitors for steels, metals and alloys, their synthesis, as well as experimental results that have highlighted these materials used for corrosion tests, the proposed mechanisms, acidic or basic media used, and the quantum chemical parameters that are correlated with their properties.

## Synthesis of the benzimidazoles with corrosion inhibition properties

**Fig. 2:**
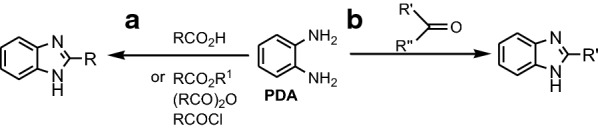
Synthesis of the benzimidazoles from 1,2-diaminobenzene (**PDA**)

Today, there are many classical benzimidazoles syntheses that have improved over the classic synthesis initially used by Hoebrecker. An extensive review of the synthesized benzimidazoles was carried out by Wright [14] and Preston [15]. Mainly, there are two methods used for synthesis of benzimidazoles: a. The reaction of 1,2-diaminobenzene (**PDA**) with carboxylic acids—Phillips–Ladenburg method in presence of 4 N HCl as catalyst; b. reaction of 1,2-diaminobenzenes with aldehydes or ketones—Weidenhagen method (Fig. 2). In the Phillips-Ladenburg reaction, also can be used: esters, acid anhydrides, acid chlorides and lactones instead of acids. Also, 2-nitroaniline in presence of a reductive agent can be used as substrate instead of **PDA** [14, 15].

Several syntheses mentioned in the literature for benzimidazoles with corrosion inhibitor properties will be given below.

## Synthesis of benzimidazoles by Phillips–Ladenburg method

**Fig. 3:**
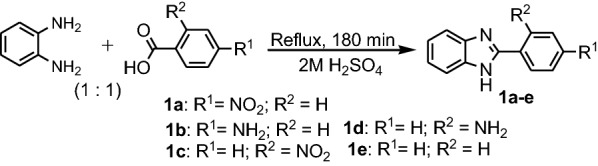
Synthesis of benzimidazoles **1a**–**e**

**Fig. 4:**
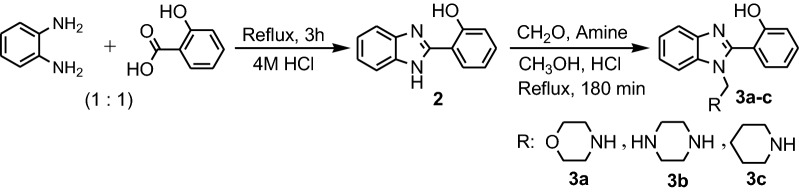
Synthesis of Mannich benzimidazole bases **3a**–**c**

Dutta et al. synthesized five benzimidazoles **1a**–**e** by refluxing **PDA** with aromatic carboxylic acids, for 3 h in presence of 2 M sulfuric acid as catalyst (Fig. 3) [16]. Yadav et al. synthesized three benzimidazole Mannich bases **3a**–**c** in two steps: i. synthesis of 2-(1*H*-benzo[d] imidazol-2-yl)phenol **2** by refluxing **PDA** with salicylic acid for 3 h with 4 N HCl and ii. synthesis of Mannich bases by reaction between **2**, formaldehyde and one secondary amine, morpholine, piperazine or piperidine under reflux in methanol for 180 min (Fig. 4) [17].

**Fig. 5:**
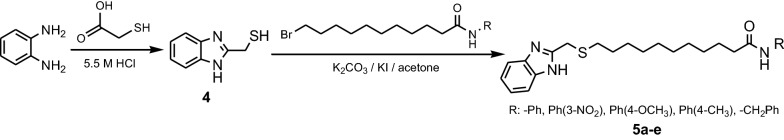
Synthesis of 2-substituted benzimidazoles **5a**–**e**

Yildirim et al. synthesized a series of 2-substituted benzimidazoles **5a**–**e** in two steps: synthesis of 1*H*-Benzo[*d*]imidazol-2-yl)methanethiol **4** by Phillips reaction; and final compounds by refluxing **4** with 11-Bromo-*N*-arylundecanamide in acetone along with potassium carbonate and a catalytic amount of sodium iodide under nitrogen atmosphere (Fig. 5) [18].

## Synthesis of benzimidazoles by Weidenhagen method

**Fig. 6:**
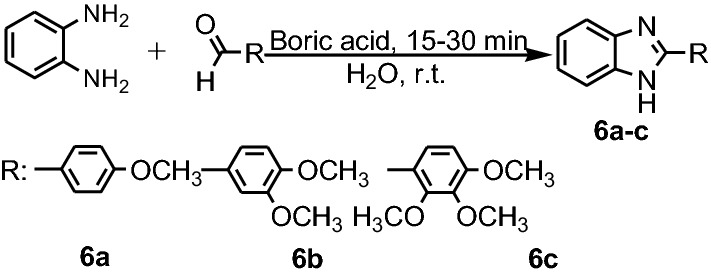
Synthesis of benzimidazoles **6a**–**c**

Singh et al. reported synthesis of benzimidazoles **6a**–**c**, starting from **PDA** and three aldehydes, in presence of boric acid as catalyst in water, at room temperature for 15–30 min (Fig. 6) [19].

## Synthesis of poli-benzimidazoles by Phillips–Ladenburg method

**Fig. 7:**
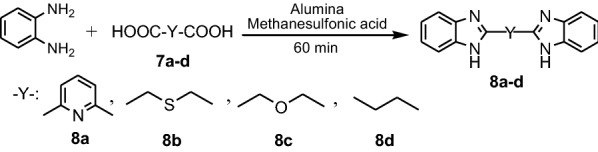
Synthesis of benzimidazoles **8a**–**d**

Dutta et al. reported synthesis of four bis-benzimidazoles **8a**–**d** by microwave irradiation method, at 180 W, starting by **PDA** and four dicarboxylic acids **7a**–**d** in presence of methanesulfonic acid and alumina (Fig. 7) [20].

**Fig. 8:**
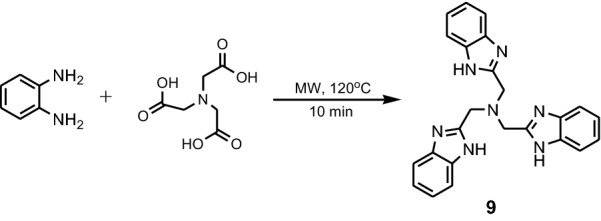
Synthesis of tris(2-benzimidazolylmethyl)amine **9**

Ibrahim et al. synthesized tris(2-benzimidazolylmethyl)amine **TBIA** by microwave heating (500 W), at 120 °C from **PDA** and 2,2′,2′′-nitrilotriacetic acid (Fig. 8) [21].

## Benzimidazoles as corrosion inhibitors in corrosive media

**Fig. 9:**
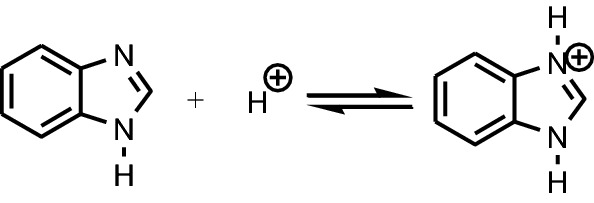
Ionization of benzimidazole in acidic medium

The chemical substances which will prevent or decrease the corrosion rate of the metal or alloy, by adding them in small quantities, in the corrosive environment, are called corrosion inhibitors [1]. It is known that the nitrogen-containing organic compounds, like amines, amides, nitriles, imines, heterocycles (triazoles, pyridines, quinolines) have been noted as corrosion inhibitors for steels during the acidizing procedure [21]. Benzimidazoles also are one of the classes of organic compounds used as corrosion inhibitors in the last decades. Benzimidazoles used as inhibitors can be considered as organic bases which are protonated in an acidic medium, predominantly at the nitrogen atom at the 3-position in the imidazole ring [22]. The cations formed are in equilibrium with the benzimidazole molecular form as show in Fig. 9.

Literature mentions various techniques used to assess the corrosion inhibition efficiency and to characterize the protective film formed on the metal surface for benzimidazoles, such as, weight loss (WL); electrochemical measurements, like electrochemical impedance spectroscopy (EIS), linear sweep voltammetry (LSW), polarization curve method or Tafel plot measurements; thermometric method; hydrogen evolution measurements; microscopy techniques, like scanning electron microscopy (SEM) or atomic force microscopy (AFM), etc. (Table 1).

**Table 1:** The inhibition efficiencies, η, of different benzimidazoles^a^ as corrosion inhibitors on the different materials in various solutions

Inhibitor (I)	Medium	Material	I_conc_ (mmol/L)	Techniques	η (%)	Refs.
Benzimidazole **1**	Deaerated 1 mol/L HCl	MS	20	EIS; TE at T = 20–60 °C	29.5–60%	[22]
2-Aminobenzimidazole **10**			20		84.0–86.8%	
2-Mercaptobenzimidazole **11**			1		93.9–97.0%	
1-Benzylbenzimidazole **12**			5		97.2–97.8%	
1,2-Dibenzylbenzimidazole **13**			1		97.4–98.2%	
2-Mercaptobenzimidazole **11**	1 M HCl	MS	50–1000	TE at 30 °C; SEM–EDX; EIS	82.6–96.5	[23]
1,8-Bis(1-chlorobenzylbenzimidazolyl)-octane **14**	0.1 M HCl	MS	0.0017–0.13	TE at 30 °C; WL; EIS; SEM	54.3–97.6%	[24]
	0.2 M HCl				55.8–97.5%	
	0.5 M HCl				57.8–97.8%	
	1 M HCl				70.0–97.9%	
1, 4-Bis-benzimidazolyl-butane **15**	0.5 M HCl	MS	0.017–0.68	EIS, TE at 30 °C; AI; WL; AFM	64.2–98.1%	[25]
*N*-(2-(1*H*-benzo[d]imidazol-2-yl)ethyl) benzamide **16**	1 M HCl	MS	0.1–1	EIS; TE at 25 °C; AI; DFT	72–97%	[26]
*N*-(2-(1*H*-benzo[d]imidazol-2-yl)ethyl)benzenesulfonamide **17**					67–96%	
*N*-((1H-benzo[d]imidazol-2-yl)methyl)benzamide **18**					56–95%	
*N*-((1*H*-benzo[d]imidazol-2-yl)methyl)benzenesulfonamide **19**					40-93%	
2-(4-Pyridil)benzimidazole **20**	1 M HCl	MS	0.1–2	EIS; WL at 25, 35 and 45 °C; AI; MD; DFT	72.4–94.05%	[27]
2-Aminomethylbenzimidazole **21**	1 M HCl	MS	0.5–0.1	TE at 25 °C; WL; AI; SEM; XPS	77.4–84.0%	[28]
Bis(2-Benzimidazolylmethyl) amine **22**					88.1–88.8%	
Tri(2-Benzimidazolylmethyl) amine **9**					91.4–93.4%	
2-Methylbenzimidazole**23**	1 M HCl	MS	0.05–0.2	WL and EI at 30, 35 and 45 °C	88.3–91.01%	[29]
2-Ethylbenzimidazole **24**					88.3–91.3%	
2-Propylbenzimidazole **25**					88.3–91.4%	
2-(2-Pyridyl)benzimidazole **26**	1 M HCl	MS	0.5–5	TE at 30 °C WL; EIS; AI	91.33–97.18%	[30]
2-Chlorobenzimidazole **27**					88.55–95.43%	
2-Bromobenzimidazole **27**					87.16–93.78%	
6-(Dodecyloxy)-1*H*-benzo[d]imidazole **29**	1 M HCl	MS	0.1–0.001	WL at 25 ± 1 °C; DFT	74.2–95.0%	[31]
2-(4-Nitrophenyl) benzimidazole **1a**	1 M HCl	MS	0.1–1	TE at 25 °C; WL at 30 °C; EIS; SEM; DFT; MD	90.3–95.9%	[16]
2-(4-aminophenyl) benzimidazole **1b**					86.1–93.9%	
2-(2-nitrophenyl) benzimidazole **1c**					82.8–90.9	
2-(2-aminophenyl) benzimidazole **1d**					79.1–89.3%	
Bis-benzimidazole **8a**	1 M HCl	MS	0.1–1	TE at 25 °C; WL at 25 °C; MD	84.0–97.0%	[20]
Bis-benzimidazole **8b**					73.0–94.5	
Bis-benzimidazole **8c**					62.0–94.0	
Bis-benzimidazole **8d**					62.0–94.0	[20]
1-Butyl-3-methyl-1*H*-benzimidazolium iodide **30**	0.5 M H_2_SO_4_	MS	0.25–5	TE at 25 °C WL; EIS; AI; SEM; AFM; DFT	90.6–98.7%	[32]
Benzimidazole **1**	1 M HCl	CS	0.05–0.25	TE at 25 °C; AI	35.5–52.0%	[33]
2-Methylbenzimidazole **23**					40.0–56.0%	
2-Mercaptobenzimidazole **11**					72.0–89.0%	
Benzimidazole **1**	0.5 M H_2_SO_4_				32.4–50.0%	
2-Methylbenzimidazole **23**					38.2–52.9%	
2-Mercaptobenzimidazole **11**					75.0–91.2%	
Benzimidazole **1**	0.5 M H_2_SO_4_+ 0.02 mM NaBr				32.4–50.0%	
2-Methylbenzimidazole **23**					38.2–52.9%	
2-Mercaptobenzimidazole **11**					75.0–91.2%	
2-Chlorobenzimidazole **27**	1 M HCl	CS	0.1–10	TE at 25 °C EIS; AI; DFT	39.4–57.1%	[2]
6-Bromo-1*H*-benzimidazole **28**					25.9–89.4%	
1*H*-Benzimidazole-5-amine **32**					34.0–74.9%	
2-Aminomethylbenzimidazole **21**					21.8–69.1%	
1*H*-Benzimidazol-5-yl-methanol **33**					26.4–73.3%	
*N*,*N*′-bis(benzimidazole-2-yl-methyl) hydroxyethylamine **34**	0.5 M HCl	CS	0.01–0.2	EIS; DFT	11.83–59.27	[34]
*N*,*N*′-bis(benzimidazole-2-yl-methyl) hydroxyethylamine **35**					41.26–69.22	
1-Octyl-2-(octylthio)-1*H*-benzimidazole **36**	1 M HCl	CS	0.01–1000 mM	EIS; PP; AI; SEM; MD	74.7–92.3	[35]
1-(2-Pyridinyl)-2-(*o*-hydroxyphenyl)Benzimidazole **37**	1 M HCl	API 5L X52 steel	0.005–0.2	EIS; SEM	67.7–91.0	[38]
1-(2-Pyridinyl)-2-(*m*-hydroxyphenyl)Benzimidazole **38**					56.8–86.2	
1-(2-Pyridinyl)-2-(*p*-hydroxyphenyl)Benzimidazole **39**					64.9–83.7	
Benzimidazole **1**	3% NaCl	AIS 316	0.025–1	TE at 24 °C	40.42–71.44%	[39]
		AIS 1010			2.41–51.21%	
2-(2-Pyridyl)benzimidazole **26**	NACE brine ID196	API X60 steel	2.56, 7.68	TE at 30 °C FTIR; EIS	46.0–67.3	[40]
2-(4-Methoxyphenyl)-1*H*- Benzo[d]Imidazole **6a**	3.5 wt % NaCl + CO_2_	J55 steel	0.176–1.407	TE at 25 °C; WL at 60 °C; EIS; XPS	56.8–94.7%	[19]
2-(3,4-Dimethoxyphenyl)-1*H*-Benzo[d] Imidazole **6b**					50.5–87.2%	
2-(3,4,5-Trimethoxyphenyl)-1*H*-Benzo[d] Imidazole **6c**					37.2–79.3%	
Benzimidazole **1**	1 M HCl	Pure Fe	1–50	TE at 25 °C; EIS. AI	47.58–51.07%	[41]
2-Aminobenzimidazole **10**					70.64–78.28%	
2-Hydroxybenzimidazole **40**					51.88–58.05%	
2-(2-Pyridil)benzimidazole **26**					62.84–69.83%	
2-Aminomethylbenzimidazole **21**	1 M HCl	Pure Fe	1–50		60.77–68.24%	[41]
2-Mercaptobenzimidazole **11**	0.1 M HCl	Al	1–100	TE at 20-60 °C. AI	60.1–80.9%	[42]
5-Methylbenzimidazole-2-thiol **41**			0.01–1		58.2–77.0%	
5-Chlorobenzimidazole-2-thiol **42**			0.01–1		47.4–78.3%	
Benzimidazole **1**	10% AcOH	6061 Al-SiCp	0.05–0.2	TE at 30–50 °C	13.29–58.56%	[43]
	20% AcOH				17.05–64.58	
	30% AcOH				10.29–58.90	
2-Mercaptobenzimidazole **11**	Aerated 0.5 M HCl	Cu	0.5	TE at 40 °C; WL at 40 °C; EIS	91.6%	[44]
2-Mercaptobenzimidazole **11**	1 M HNO_3_	Cu	0.005–1	WL at 25, 35 and 45 °C	27.5–91.5%	[45]
2-(methylthio)benzimidazole **43**					28.5–92.5%	
2-{[(6-Nitro-1*H*-benzimidazol-5-yl)imino]methyl}phenol(**44**	0.4 M NaCl + 0.1 M NaOH	Cu, brass	1	TE at 25 °C; EIS	91.0; 97.4	[46]
1-{[(6-Nitro-1H-benzimidazol-5-yl)imino]methyl}naphthalen-2-ol **45**					71.0; 92.0	
2-Mercaptobenzimidazole **11**	0.1 M HCl	Zn	0.063–0.5	TE at 30 °C; WL; AI; SEM	68.5–89.7%	[47]
Ethyl-2-(benzimidazol-2-yl-thio)acetate **47**			0.063–0.5		75.2–93.0%	
2-Hydroxybenzimidazole **40**			0.063–0.5		57.4–85.3%	
2-Hydroxy-5-nitro-benzimidazole **46**			0.05–0.2		55.6–82.5%	

*TE* Tafel extrapolation, *WL* weight loss, *EIS* electrochemical impedance spectroscopy, *MS* mild steel, *CS* carbon steel, *SS* stainless steel, *η* inhibition effectiveness, *EIS* electrochemical impedance spectroscopy, *CR* corrosion rate, *AI* adsorbtion isotherm, *SEM* scanning electron microscopy, *AFM* atomic force microscopy, *FTIR* Fourier transform infrared, *OP* optical profilometry, *XPS* X-ray Photoelectron Spectroscopy (XPS), *MD* molecular dynamics simulation, *EDX* energy-dispersive X-ray spectroscopy, *DFT* Density Functional Theory

^a^The structures of the benzimidazoles are found in Figs. 2, 3, 4, 5, 6, 7, 8, 9, 10, 11

Benzimidazoles are used as corrosion inhibitors on various acids, such as hydrochloric acid (HCl), sulfuric acid (H_2_SO_4_), acetic acid (CH_3_COOH), but most commonly used is HCl at 0.1 M–1 M concentration [13]. Table 1 shows the name of the benzimidazoles tested as corrosion inhibitors, the acid media used, the material, the inhibitor concentration, test conditions, testing techniques and inhibition efficiency η. The inhibition efficiency (IE) can be calculated using the Eq. (1):

1 $$ \eta \% = \frac{{\nu_{0} - \nu }}{{\nu_{0} }}100 $$

where $\nu_{0}$ and $\nu$ are the corrosion rate in the absence and presence of inhibitor, respectively [23].

## Benzimidazoles as corrosion inhibitors for mild steel (MS)

### For hydrochloric acid (HCl) medium

**Fig. 10:**
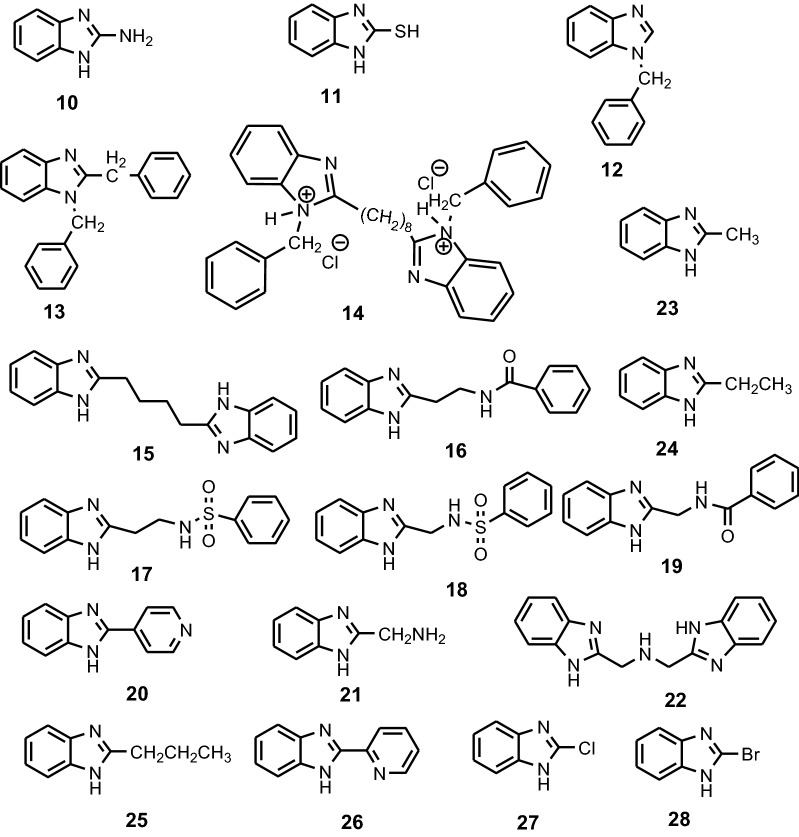
Chemical structures of the benzimidazoles **10**–**28**

Popova et al. reported that benzimidazole, 2-aminobenzimidazole, 2-mercaptobenzimidazole, 1-benzylbenzimidazol and 1,2-dibenzylbenzimidazole **10**–**13** (Fig. 10) have pronounced corrosion inhibition properties at all temperatures for MS in deaerated 1 mol/L HCl solution [22] (Table 1). The inhibiting behaviour appears as consequence of energetic effect and blocking of the active surface atoms, leading to the decrease of the surface concentration of the hydrogen ions and the increase of hydrogen evolution overpotential. Mahdavian et al. studied the effect of 2-mercaptobenzimidazole **11** on the corrosion of MS in 1 M HCl solution by polarization and electrochemical impedance spectroscopy (EIS) [23]. Both anodic and cathodic current densities were decreased with increasing concentration from 0.05 mM to 1 mM, indicating that **11** suppressed both the anodic and cathodic reactions through adsorption on the MS surface, suggesting that **11** act as mixed type corrosion inhibitor. The results of SEM–EDX showed presence of sulfur on the MS surface confirming the adsorption of **11** on the MS surface as showed by the EIS measurements. Wang et al. shows that 1,8-bis(1-chlorobenzylbenzimidazolyl)-octane **14** (Fig. 10) acted as an excellent inhibitor via strongly chemical adsorption onto MS surface to suppress simultaneously both anodic and cathodic processes according to the Langmuir adsorption isotherm [24]. They found that increasing concentration of inhibitor and HCl lead to increasing of inhibition efficiencies (IEs) and proposed an inhibition mechanism. Wang et al. found that 4-bis-benzimidazolyl-butane **15** inhibits both anodic and cathodic processes of mild steel in 0.5 M HCl solution and IE enhances with the increase of concentration of inhibitor. AFM images show the mean roughness of the.

**Fig. 11:**
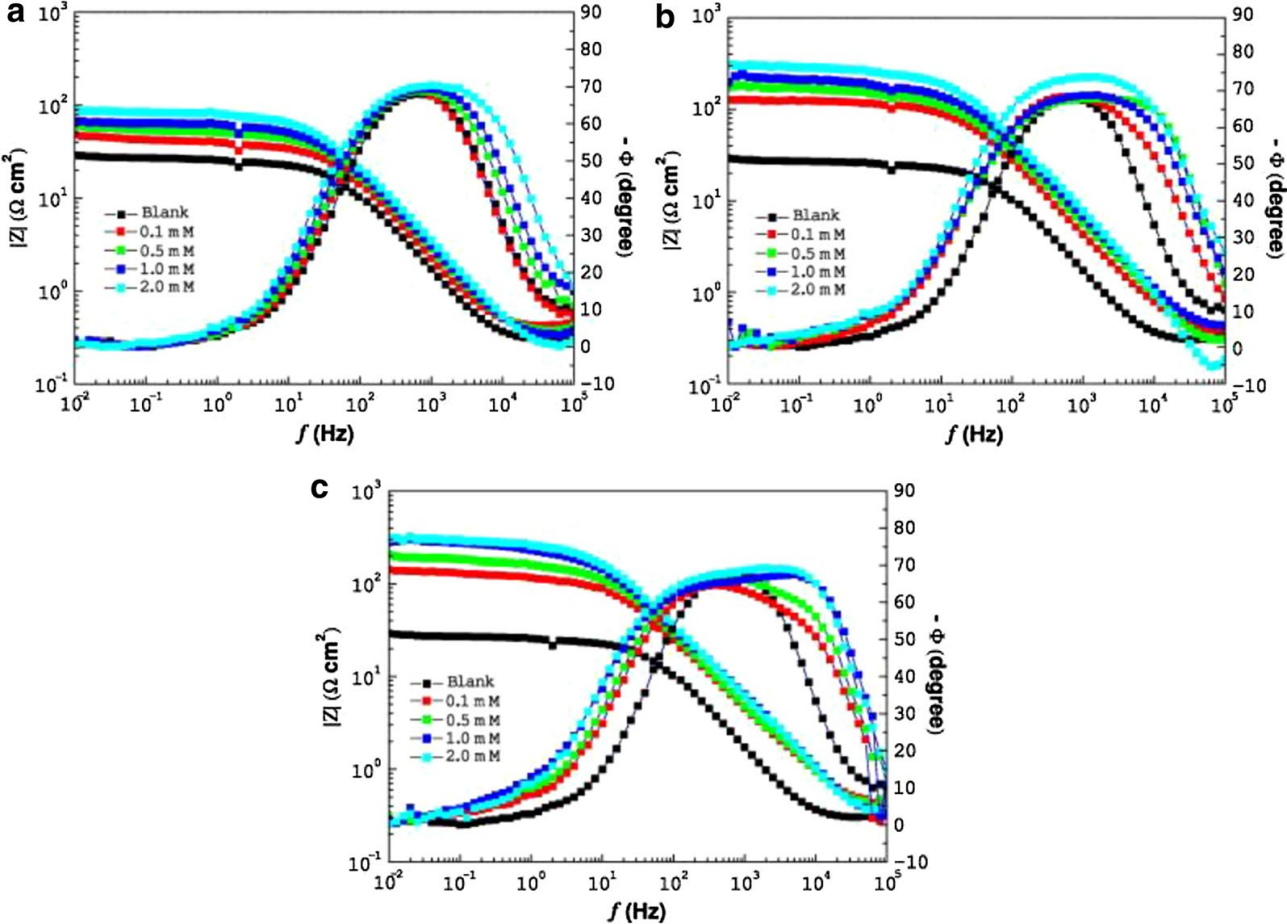
Bode plots for mild steel in 1.0 M HCl without and with different concentrations of inhibitors at 25 °C for compounds: **a 21**, **b 22**, and **c 9** (Reproduced with permission from Elsevier, Ref. [28])

**Fig. 12:**
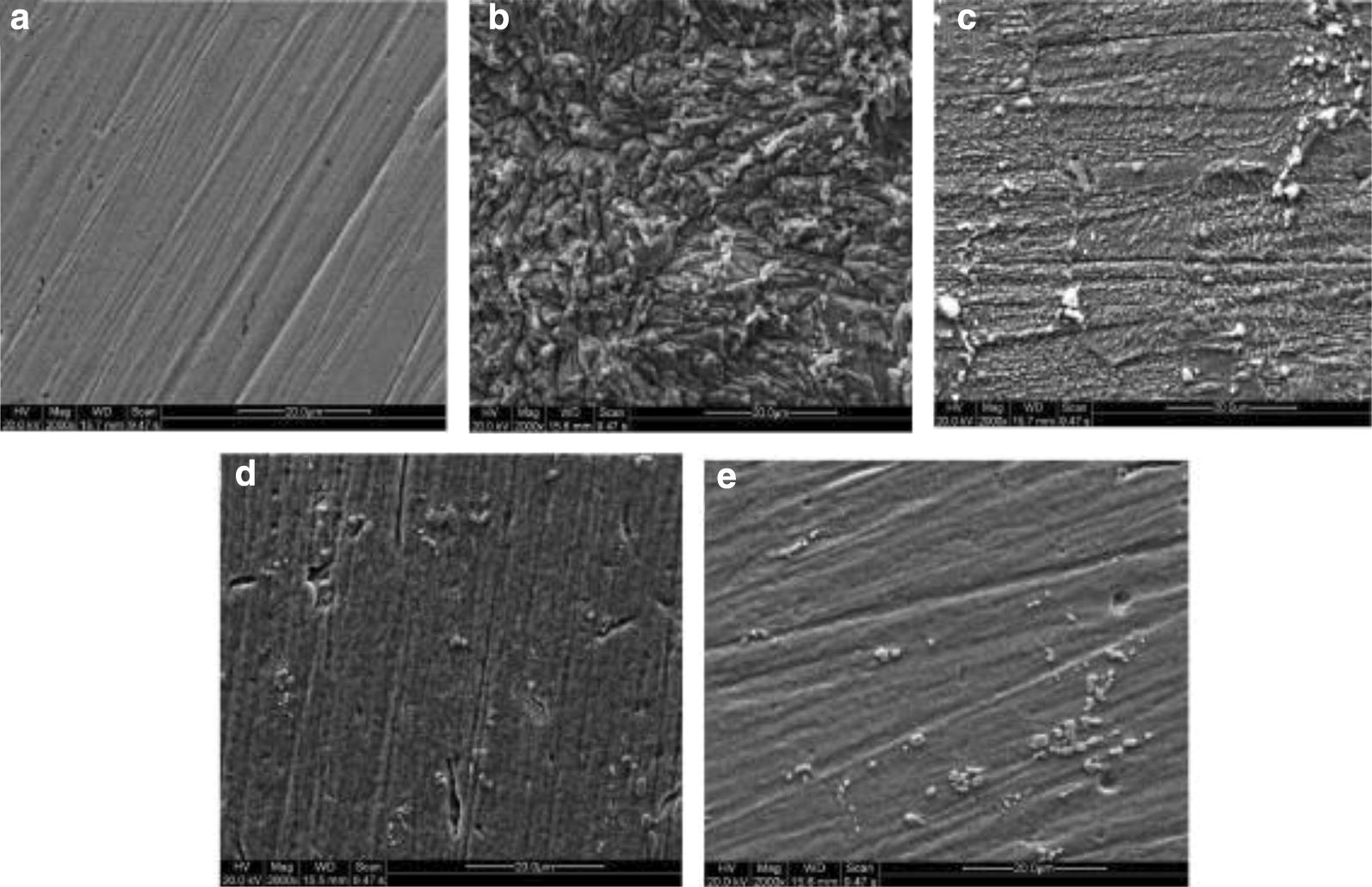
SEM images for mild steel surface: **a** before immersion, **b** blank, **c 21**, **d 22**, and **e 9** (Reproduced with permission from Elsevier, Ref. [28])

**Fig. 13:**
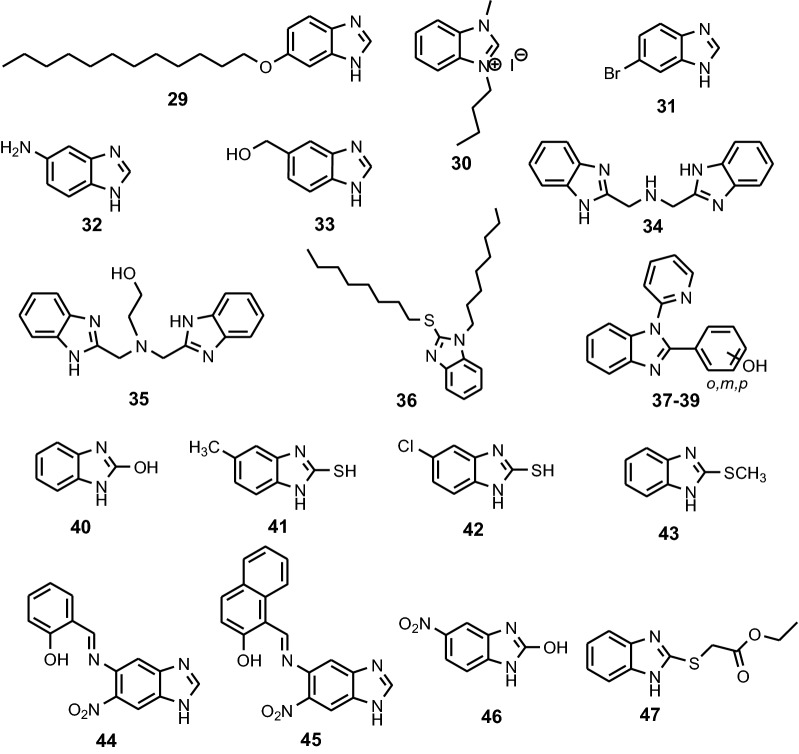
Chemical structures of the benzimidazoles **29**–**47**

The protective film formation of inhibitor **15** protects MS from corrosion. Thus, in experimental conditions, as consequence of the 0.5 M HCl solution attack, MS surface is 42.18 nm, in uninhibited solution, while the roughness of 12.69 nm is obtained in the presence of benzimidazole **15**. The adsorption of **15** on MS surface obeys Langmuir adsorption isotherm. Dutta et al. applied electrochemical techniques and found that four benzimidazoles **16**–**19** act as mixed type inhibitors in 1 M HCl solution on mild steel [26]. From Temkin adsorption isotherm studies, it may be argued that inhibitors adsorb spontaneously on the MS surface. It was proposed that inhibitors are adsorbed parallel to the metal surface through the benzimidazole moiety, considering electron density in the HOMO level mostly distributed over this planar moiety. Zhang et al. found that 2-(4-pyridyl)benzimidazole **20** acts as a mixed-type inhibitor with predominant cathodic effectiveness, being a good inhibitor for MS corrosion in 1.0 M HCl [27]. Presence of compound **20** inhibits the reduction of H^+^ ions, by immobilization of the reactive centers from the MS surface, without changing the process mechanism, as can be seen by the Tafel lines. Inhibition efficiency (IE) depends on the temperature and the concentration of the acid solution, and also increases with increasing inhibitor concentration. Tang et al. reported the inhibition properties for the corrosion of MS in 1 M HCl of 2-aminomethylbenzimidazole **21**, bis(2-benzimidazolylmethyl) amine **22** and tris(2-benzimidazolyl methyl) amine **9** [28]. The IEs of the three compounds decreasing the order: **9** > **22** > **21**, for the same concentration, showing that the IE increases with the increase in the number of benzimidazole scaffold in the molecules. From the potentiodynamic polarization (PP) curves (Fig. 11), all these inhibitors exhibit stronger inhibitive effect on the cathodic reaction than on the anodic one, indicating that the compounds act as mixed type inhibitors for reaction of mild steel in 1.0 M HCl. SEM images (Fig. 12) confirm the effectiveness of these inhibitors as the surface of MS in the uninhibited solution is seriously damaged in comparison with that before immersion. In the presence of the inhibitors, the damage of the steel surfaces is significantly reduced. Ramya et al. found that 2-methylbenzimidazole **23**, 2-ethylbenzimidazole **24** and 2-propyl-benzimidazole **25** (Fig. 10) are effective CIs for the corrosion of MS in 1 M HCl [29]. The adsorption of inhibitors is neither typical physisorption, nor chemisorption but it is complex mixed type, as resulted from the values of standard free energy of adsorption $\Delta G_{ads}^{0}$. Lgaz et al. reported the inhibition effects on MS in 1 M HCl of 2-(2-pyridyl)benzimidazole **26**, 2-chlorobenzimidazole **27** and 2-bromobenzimidazole **28** [30]. These compounds have good IE, which increases with the inhibitor concentration and follows the order: **26** > **28** > **27**. Langmuir adsorption isotherm suggest that these derivatives are strongly adsorbed on the mild steel surface and PP study shows that the studied compounds are mixed type inhibitors. Zhang et al. reported that 6-(dodecyloxy)-1*H*-benzo[d]imidazole **29** (Fig. 13) exhibits effective inhibition for corrosion of MS in 1 M HCl medium [31]. Table 2 depicts the corrosion rate of MS and inhibition efficiency from weight loss measurements in the absence and presence of benzimidazole **29**. It is obviously that the corrosion rate of MS decreases significantly and the inhibition efficiency increases with increasing inhibitor concentration.

**Table 2:** Corrosion rate and inhibition efficiency in the absence and presence of 6-(dodecyloxy)-1*H*-benzo[d]imidazole **29** as inhibitor in 1 M HCl solution. reproduced with permission from Elsevier, Ref. [31]

C(M)	ν (gm^−2^ h^−1^)	η(%)	θ
0	4.8 ± 0.02	–	–
10^−6^	1.24 ± 0.16	74.2	0.742
2 10^−6^	1.06 ± 0.23	77.9	0.779
4 10^−6^	0.81 ± 0.04	83.1	0.831
8 10^−6^	0.39 ± 0.03	91.9	0.919
10^−5^	0.35 ± 0.01	92.7	0.927
10^−4^	0.24 ± 0.01	95.0	0.950

**Fig. 14:**
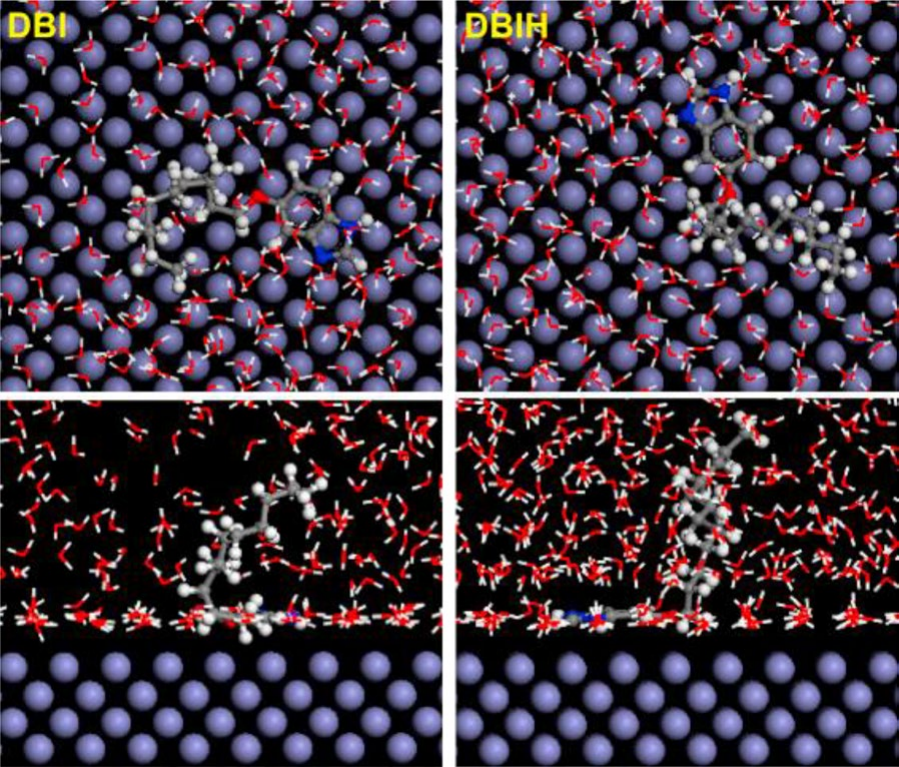
Equilibrium configurations of **29** (DBI) and its protonated form (DBIH) in aqueous solution. Top: topview, bottom: sideview (Reproduced with permission from Elsevier, Ref. [31])

Following the theoretical study performed on compound **29** (Fig. 14), it was determined that its structural “key” is the non-polar long chain, which blocks the action of the erosive solution, and is absorbed on MS by electron donation to the vacant d-orbital. Therefore, the non-protonated benzimidazole **29** (DBI), less polar, is a much more effective inhibitor against erosive species on the iron surface, compared to its protonated species (DBIH), surrounded by a thick layer of water. Dutta et al. studied the inhibition properties of five benzimidazoles **1a**–**e** (Fig. 3) towards MS in 1 M HCl [16]. A strong decrease in corrosion rate is determined by the inhibitors **1a**–**e** in acidic solution, as can be seen in polarization curves. Studies show that *para*-substituted derivatives are more potent anticorrosive agents than *ortho*-substituted compounds, and nitro-derivatives are better inhibitors than amine homologues. The mechanism of action of the benzimidazoles as inhibitors was determined by electrochemical impedance spectroscopy (EIS). Nyquist and Bode impedance plots indicate the physical adsorption of benzimidazoles on the metal surface, which prevents reactions at the metal-solution interface. Also, optimized molecular geometry and electron distribution in the frontier molecular orbitals provided valuable information on charge transfer during adsorption. Dutta et al. reveal that four bis-benzimidazoles derivatives **8a**–**d** (Fig. 7) possess good inhibition efficiency for mild steel in 1 M HCl [20]. The inhibition efficiencies of the benzimidazoles towards prevention of corrosion of mild steel in 1 M HCl follow the order **8a **> **8b **> **8c **> **8d**, and, more important, their effectiveness is maintained for an exposure time of as long as 96 h. EIS study indicates that the extent of adsorption increases with concentration of the inhibitors and thus provides better barrier towards charge transfer reactions at the metal–solution interface. Quantum mechanical studies show a series of factors, like stereochemistry of the molecule, presence of heteroatoms, π electron density and charge on individual atom important for corrosion inhibitors.

### For sulfuric acid (H_2_SO_4_) medium

**Fig. 15:**
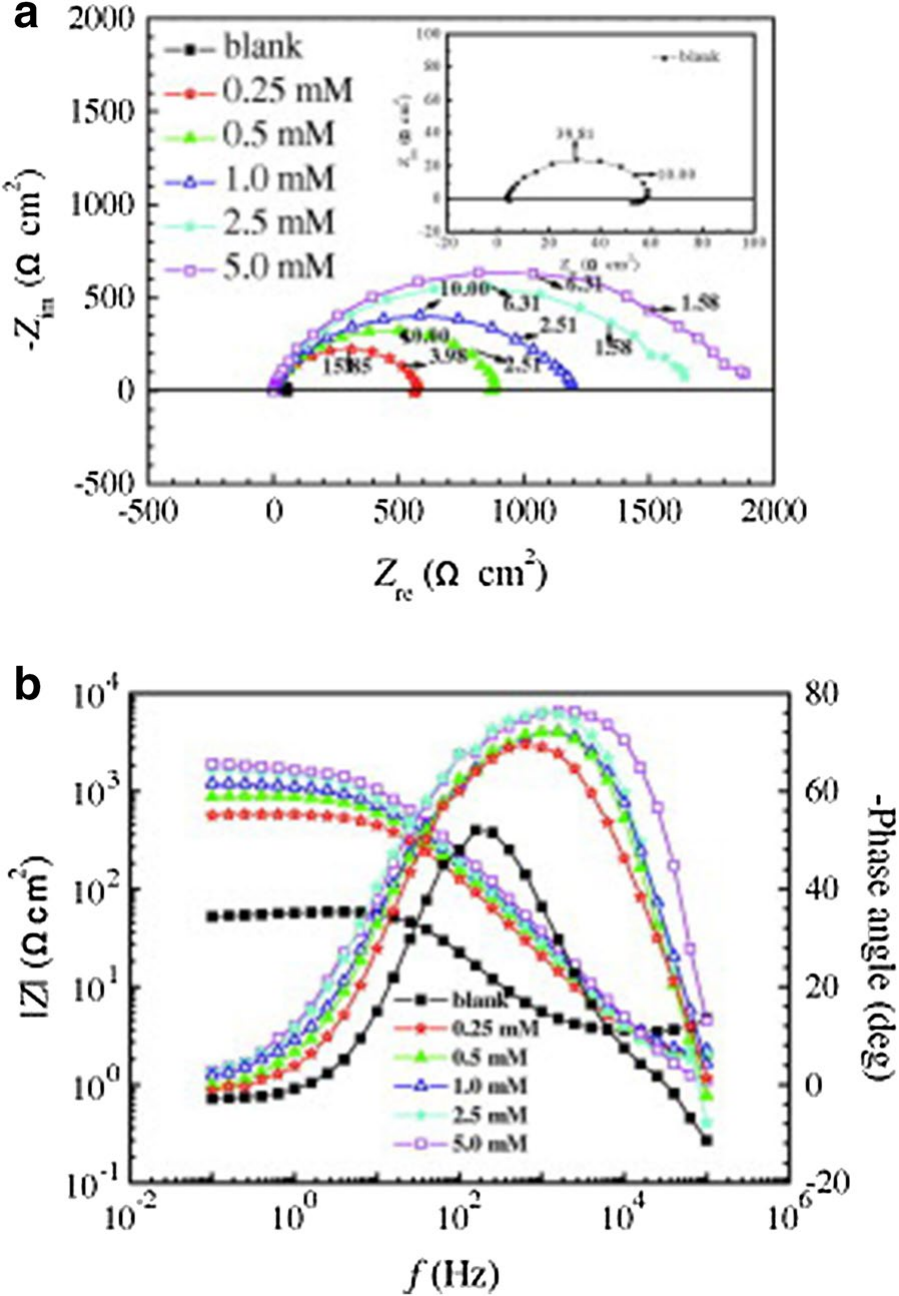
EIS for mild steel in 0.5 M H_2_SO_4_ solution without and with different concentration of 1-buthyl-3-methyl-1*H*-benzimidazolium iodide 30 at 298 K: **a** Nyqusit plots, and **b** Bode plots (Reproduced with permission from Elsevier, Ref. [32])

Zheng et al. revealed that **30** is a good inhibitor for mild steel in 0.5 M H_2_SO_4_ solution and IE increases with the increasing concentration of **30** (Fig. 13) [32]. Addition of inhibitor **30**, at different concentrations, at a temperature of 298 K, to a solution of 0.5 M H_2_SO_4_, led to a relatively small inhibition of the corrosion effect on MS, which is shown in the Nyquist (a) and Bode (b) plots (Fig. 15). The only exception to visible improvement of corrosion inhibition is visualized on the diameter of high frequencies capacitive loop which markedly increases with enhancing inhibitor concentration. In this case, the gradual disappearance of the low-frequency loop is observed, that is, a “degradation” phenomenon of impedance spectroscopy. A strong interaction between inhibitor **30** and the MS surface in 0.5 M H_2_SO_4_ is revealed by the positive values of $\Delta S_{ads}^{0}$, negative values of $\Delta G_{ads}^{0}$ and high values of adsorbtion constants K_ads_, as seen in Table 3.

**Table 3:** The thermodynamic parameters for mild steel in 0.5 M H_2_SO_4_ solution containing different concentration of 1-butyl-3-methyl-1*H*-benzimidazolium iodide **30** at different temperature. Reproduced with permission from Elsevier, Ref. [32]

Temperature (K)	K_ads_ (L/mol)	$\Delta G_{ads}^{0}$ (kJ/Mol)	$\Delta H_{ads}^{0}$ (kJ/Mol)	$\Delta S_{ads}^{0}$ (J K^−1^ Mol^−1^)
298	16,516	− 34.03	− 32.89	3.83
308	13,100	− 34.58	–	5.49
318	7864	− 34.35	–	4.59
328	5053	− 34.22	–	4.06

Compound **30** proved to be a mixed type corrosion inhibitor because polarization measurements inhibit both the anodic and cathodic processes of the corrosion of MS in 0.5 M H_2_SO_4_ solution. DFT data show that cation **30** is adsorbed on the MS surface by benzimidazole ring using a flat mode.

### Benzimidazoles as corrosion inhibitors for carbon steel (CS)

**Fig. 16:**
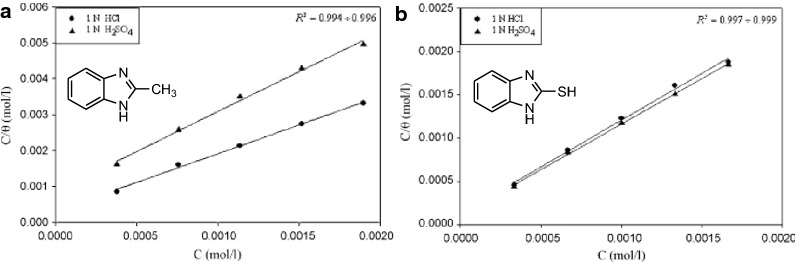
Langmuir adsorption isotherm of benzimidazoles **23** (**a**) and **11** (**b**) in 1 N HCl and 1 N H_2_SO_4_ solutions at 25 °C (Reproduced with permission from Elsevier, Ref. [33])

**Fig. 17:**
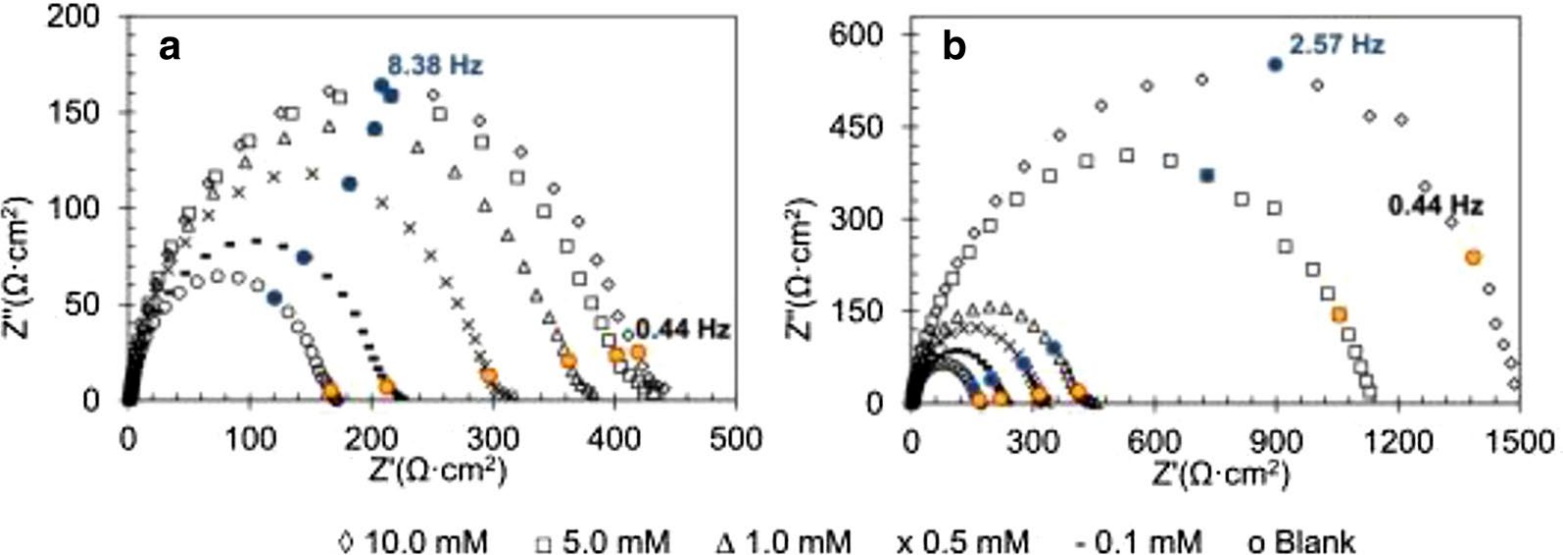
Nyquist plots of the best and worst inhibitor from benzimidazole derivatives: **a 32**, **b 28**. Blue points correspond to frequency where imaginary impedance is highest at inhibitor concentration 10.0 mM (Reproduced with permission from Elsevier, Ref. [2])

**Fig. 18:**
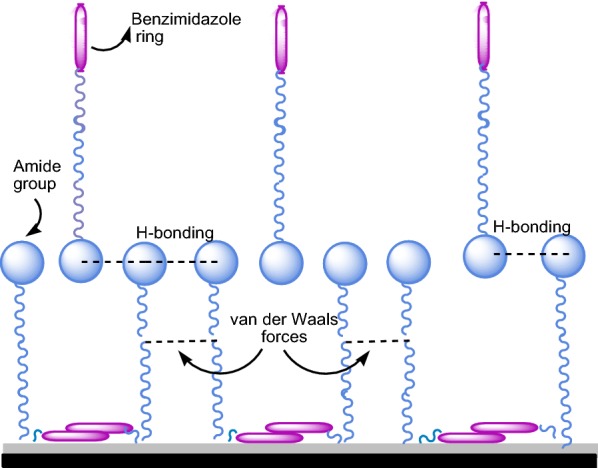
Schematic representation of the inhibitory effect by formation of the secondary molecular layer on the CS surface

Aljourani et al. studied the inhibition characteristics of benzimidazole **1**, 2-methylbenzimidazole **23** and 2-mercaptobenzimidazole **11** for CS in HCl and H_2_SO_4_ solutions using PP measurements [33]. The straight line of Langmuir absorption isotherms (Fig. 16) shows that the inhibition of CS in acid solutions by benzimidazoles is an adsorptive process. This isotherm assumes that the adsorbed molecules occupy only one site and there are no interactions between the adsorbed species [33]. Also, synergistic effects of bromine ion on the sulfuric acid and the comparing with the behaviour in hydrochloric acid and sulfuric acid elucidated the dominant active form of the inhibitors (molecular or ionic) during the adsorption process. It was found that the order of IE is **11 **> **23** > **1** in all solutions. The corrosion inhibition of **1** and **23** in both acidic media is due to the physisorption (adsorption of cationic forms), while corrosion inhibitions of **11** is due to the chemisorption of the molecular form. Guttierez et al. studied five benzimidazoles as potential corrosion inhibitors of CS in 1 M HCl, 2-chlorobenzimidazole **27**, 6-bromo-1*H*-benzimidazole **31**, 1*H*-benzimidazole-5-amine **32**, 2-amino-methyl benzimidazole **21** and 1*H*-Benzimidazol-5-yl-methanol **33** [2]. From the Nyquist plots, it is clear that the presence of the substituents in the structure of the molecule has an effect on the inhibition properties (Fig. 17). The authors concluded that differences in inhibition properties were certainly a consequence of presence of substituents and their position have a significant effect on the protection on steel. According to the mathematical model proposed the properties as electronegativity, aromaticity, volume and the charge of nitrogen atoms are associated with the corrosion inhibition efficiency. Yildirim et al. investigated the inhibition properties of five long-chain benzimidazoles **5a**–**5e** for CS in H_2_SO_4_ (Fig. 5). The 3D optical profilometer images of the metal to acidic medium without inhibitor show to corrosion damages on the metal surface, while the treatment of the metal with inhibitor prevents the corrosion of the surface [18]. The compounds exhibited the highest inhibition at 0.15 mM inhibitor concentration. Authors proposed an upright position of the inhibitor in the metal surface which provides maximum protection as is shown in Fig. 18. The inhibitor is absorbed on the metal surface through the sulfur atom and π–π interactions between aromatic rings. Intermolecular hydrogen bonds formed via amide groups, increase the protective layer. Increasing the inhibitory effect due to increased in inhibitor concentrations may indicate that secondary molecular bi-layer may occur via hydrogen bonds and Van der Waals forces. Garcia-Ochoa et al. studied the corrosion inhibition properties of **34** and **35** (Fig. 18) on CS in 0.5 M HCl medium by electrochemical and theoretical methods. Here, the corrosion rates (Table 4) were measured after the metal surface exposure to corrosive medium for 4.0 h in the presence of compounds **34** and **35** at different concentrations. It is observed that, as the concentration of inhibitors increases, the corrosion rate decreases continuously, while the corrosion potential does not suffer significantly. This indicates that the corrosion inhibition mechanism on the metal surface is an adsorption process that prevents both anodic and cathodic reactions. The data show that ligands form an adsorption layer over an iron surface, obeying the Langmuir isotherm ($\Delta G_{ads}^{o}$ of − 32.96 kJ mol^−1^); the value is in the range − 20 ÷ − 40 kJ mol^−1^, belonging to a conversion stage of physical adsorption to chemical adsorption or a comprehensive adsorption [34]. The efficiency of inhibitors increases with increasing their concentration in the corrosion medium.

**Table 4:** Electrochemical polarization data of the inhibitors. Reproduced with permission from Elsevier, Ref. [34]

Conc (mM)	β_a_ (mV dec^−1^)	β_c_ (mV dec^−1^)	E_corr_ (V)	I_corr_ (A/cm^2^)	θ	%E
Compound **34**						
0.0	194	133	− 0.5340	5.89 × 10^−4^	–	–
0.01	211	135	− 0.5284	5.194 × 10^−4^	0.1183	11.83
0.03	241	133	− 0.5470	3.977 × 10^−4^	0.3249	32.49
0.07	229	115	− 0.5472	3.577 × 10^−4^	0.3928	39.28
0.2	176	91	− 0.5433	2.399 × 10^−4^	0.5927	59.27
Compound **35**						
0.0	194	133	− 0.5340	5.89 × 10^−4^	–	–
0.01	151	117	− 0.5184	3.46 × 10^−4^	0.4126	41.26
0.03	153	112	− 0.5309	3.29 × 10^−4^	0.4415	44.15
0.07	171	119	− 0.5332	2.881 × 10^−4^	0.5109	51.09
0.2	166	95	− 0.5296	1.813 × 10^−4^	0.6922	69.22

El-Hajjaji et al. observed that both cathodic and anodic current densities decrease in presence of **36** [35], in the acidic solution, which indicate the adsorption of the inhibitor molecules onto the active sites of the CS surface [36], and suggests the inhibitor influence in the anodic metal dissolution reaction, as well as on the cathodic reaction of hydrogen evolution [37]. The successive increase in the inhibitor concentration resulted in a more pronounced inhibition behavior. The inhibitory behavior was attributed to the nitrogen atoms of the imidazole ring and the sulphur atom present in the alkyl chain of the inhibitor molecule.

## Benzimidazoles as corrosion inhibitors for other steels

### Benzimidazoles as corrosion inhibitors for acidic media

1-(2-Pyridinyl)-2-(*o*, *m*-, *p*-hydroxyphenyl) benzimidazoles **37**–**39** were studied as corrosion inhibitors in API 5L X52 steel/HCl 1 M corrosion system by Rodriguez-Clemente et al. [38]. It is proved that the inhibitory action depends on the concentration of the compound, double-layer capacitance and resistance to charge transfer. The protective, stable and uniform efficiency of the inhibitor was experimentally proved by SEM micrograph, in the presence of the inhibitor at an optimum concentration. Yadav et al. synthesized and assessed for the corrosion inhibition potentials on N80 steel in 15% HCl solution compounds **3a**–**c** (Fig. 4). Basic information on the interaction between the organic inhibitors and the N80 steel surface were obtained from various adsorption isotherms. Large values of K_ads_ obtained for all the three inhibitors indicate more efficient adsorption and hence better corrosion inhibition efficiency. All compounds show good inhibition efficiencies and the IE increases on increasing concentration of the inhibitors and decreases with increasing temperature. IE decreases in the order: **3c **> **3a** > **3b** [17]. SEM and AFM images confirmed the adsorption of the inhibitors on the steel surface while FTIR and UV–VIS spectra established the interactions between the inhibitors and N80 steel.

### Benzimidazoles as corrosion inhibitors for salt media

**Fig. 19:**
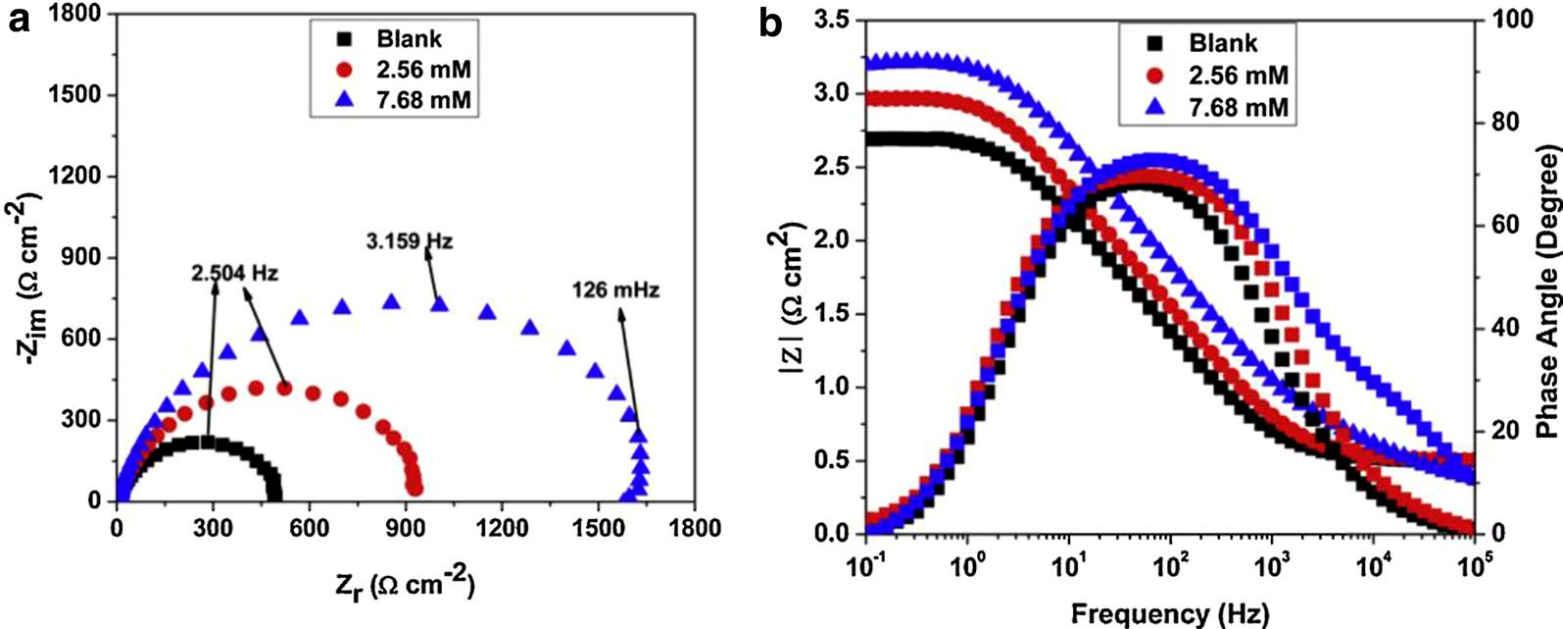
**a** Nyquist plots and **b** Bode plots for X60 steel during static CO_2_ corrosion in NACE ID196 brine without and with 26 as inhibitor (Reproduced with permission from Elsevier, Ref. [40])

**Fig. 20:**
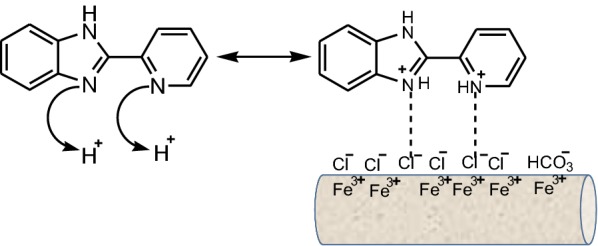
Schematic representation of the interaction between 2-(2-Pyridil)-benzimidazole **26** and API X60 steel surface during CO_2_ corrosion inhibition in the NACE brine solution

Moreira et al. reported that benzimidazole **1** possess best corrosion inhibition result for AISI 316 steel and AISI 1010 steel in 3% aqueous solution NaCl using inhibitor concentration of 0.1 mM, and the IE values were ~ 71% and ~ 51%, respectively [39]. Electrochemical studies have shown that in the presence of an inhibitor at concentration of 0.025 ÷ 1 mM, there was an increase of the polarisation resistance of both steels, indicating that there was an increase in corrosion resistance. Onyeachu et al. assessed the performance of 2-(2-pyridyl)benzimidazole **26** (Fig. 10) as inhibitor for the CO_2_ corrosion of X60 steel in NACE brine ID196 (contained 3.5 wt % NaCl, 0.305 wt% CaCl_2_·2H_2_O and 0.186 wt% MgCl_2_·6H_2_O prepared with double distilled water) with electrochemical methods [40]. The extended Nyquist semicircle plots (Fig. 19) shows the molecules of the compound **26** adsorbed on the steel surface lead to a barrier that isolates against the brine solution. Under static and hydrodynamic conditions, inhibitor **26** behaves very well during CO_2_ corrosion, but not so well at high rotation speed, when the inhibition efficiency is decreased. The best adsorption of the inhibitor **26** is realized by the formation of the pyridinium ion after the protonation of 2-pyridyl nitrogen. The proposed mechanism for this interaction is shown in Fig. 20. Singh et al. reported that benzimidazoles **6a**–**6c** (Fig. 6) are good inhibitors in 3.5% NaCl solution saturated with carbon dioxide at 60 °C for J55 steel [19]. The potentiodynamic polarization measurement supports the mixed mode of inhibitors with predominantly cathodic effects. Both experimental and theoretical investigations suggest that increasing the number of methoxy groups improve corrosion protection ability of the inhibitors, therefore, **6c** with three methoxy groups is the best inhibitor. The DFT study confirms that the imine nitrogen is the most preferred site for protonation.

## Benzimidazoles as corrosion inhibitors for other metals

### Benzimidazoles as corrosion inhibitors for iron

Khaled found that benzimidazole **1**, 2-aminobenzimidazole **10**, 2-hydroxybenzimidazole **40**, 2-(2-pyridil) benzimidazole **26** and 2-aminomethylbenzimidazole **21** (Figs. 10, 13) inhibit the corrosion of pure iron in 1 M HCl solutions at 25 °C. He found that the IE increases with increasing electron donating ability of these molecules according to the order, **10** > **26** > **21** > **40 **> **1** [41]. Results obtained from potentiodynamic polarization indicated that the benzimidazole inhibitors are mixed-type inhibitors. This order can be explained by the nature of substituents on benzimidazole ring, namely, the most effective are those containing nitrogen, and among them, **10** most efficiently because the electron donating effect of the –NH_2_ group which contributes to increasing the availability of the electrons π in benzimidazole ring and attachment on the iron surface by the free amino group.

### Benzimidazoles as corrosion inhibitors for aluminum and its alloys

Bereket et al. investigated the effect of 2-mercaptobenzimidazole **11**, 5-methyl-benzimidazole-2-thiol **41** and 5-chlorobenzimidazole-2-thiol **42** on the corrosion of aluminum in 0.1 M HCl by a PP technique at 20–60 °C [42]. It was reported that the IE of these compounds has the following order: **11** > **41** > **42**. Corrosion potentials shifted to more negative values and corrosion currents decreased as the concentrations of these organic compounds was increased. These results show that the inhibitive action of all compounds was due to adsorption on the cathodic sides. Authors found that the inhibitive action of benzimidazoles was mainly due to adsorption on the metal surfaces, which show parallelism with the calculated total negative charge of each molecule. Chacko et al. used benzimidazole **1** at different concentrations as corrosion inhibitor for the 6061 Al–SiC_p_ composite on 10–30% concentrations of acetic acid and at different temperatures [43]. The maximum efficiency of inhibition obtained using **1** as inhibitor was 64.58%. IE increases with temperature and E_a_ (activation energy) values obtained for inhibited solutions are smaller than that obtained in the uninhibited solution which suggested that **1** is adsorbed on the composite surface by mixed adsorption, where chemisorption is predominant.

### Benzimidazoles as corrosion inhibitors for copper and its alloys

**Fig. 21:**
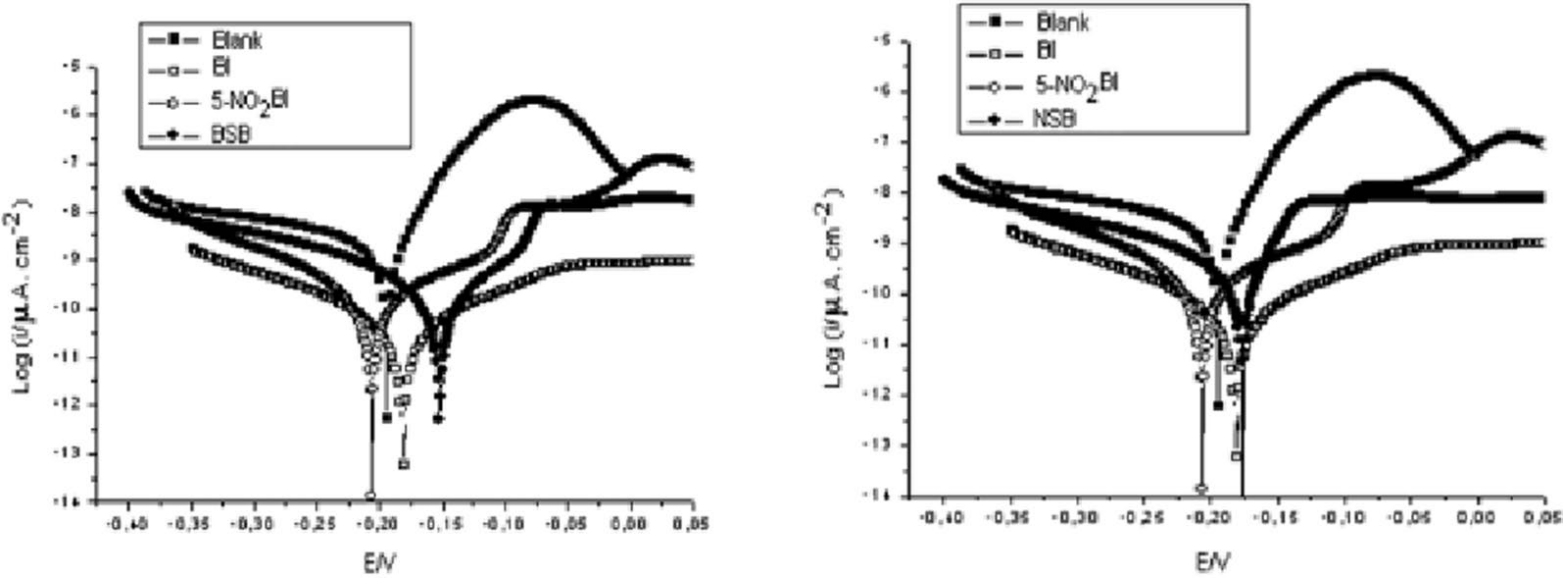
Polarization curves for copper in 0.4 M NaCl + 0.1 M NaOH solution of benzimidazoles **44** and **45** (Ref. [46])

Zhang et al. reported that 2-mercaptobenzimidazole **11** has inhibitory effects on anodic dissolution of copper in 0.5 mol/L HCl solution and that the introduction of a mercapto group to a heterocyclic compound improves its inhibitory effect [44]. Potentiodynamic polarization results revealed that **11** acted as anodic inhibitor and quantum chemical calculation shows higher energy levels of HOMO and LUMO and a larger π-electron density. Niamien et al. studied corrosion behaviour of copper in 1 M nitric acid in the presence of two CIs, 2-mercaptobenzimidazole **11** and 2-(methylthio)benzimidazole **43** (Fig. 11) via WL method and quantum chemical approaches. It was found that the inhibition efficiencies increase with increasing concentration and increasing temperature [45]. It has been found that the compounds adsorb onto copper according to the modified Langmuir adsorption isotherm and the kinetic/thermodynamic isotherm of El-Awady. Ozbay et al. reported good corrosion efficiencies for two Schiff bases 2-{[(6-nitro-1*H*-benzimidazol-5-yl)imino]methyl}phenol **44** and 1-{[(6-nitro-1*H*-benzimidazol-5-yl-imino]methyl} naphthalene-2-ol **45** on copper and brass (Cu40Zn60) in alkaline media, of 92.0% and 97.4% respectively [46]. The effect of benzimidazole derivatives on the impedance behaviour of copper and brass in alkaline media at 25 °C shows a semi-circle response. Electrochemical parameters calculated from polarization measurements on copper and brass in solution of (0.4 M NaCl + 0.1 M NaOH) without and with 10^−3^ M of benzimidazole derivatives at 25 °C are given in Table 5. Polarization curves for copper in 0.4 M NaCl + 0.1 M NaOH solution of benzimidazoles **44** and **45** are given in Fig. 21.

**Table 5:** Electrochemical parameters calculated from polarization measurements on copper and brass in 0.4 M NaCl + 0.1 M NaOH without and with 10^−3^ M of benzimidazole derivatives at 25 °C. Reproduced with permission from Elsevier, Ref. [46]

	E_corr_ Ag/AgCl (V)	− β_c_ (mV dec^−1^)	-β_a_ (mV dec^−1^)	I_corr_ (μA cm^−2)^	IE (%)
Copper					
Blank	− 0.194	324	286	41.0	–
**44**	− 0.153	206	196	3.67	91.0
**45**	− 0.168	219	291	12.0	71.0
Brass					
Blank	− 0.437	483	136	118.0	–
**44**	− 0.225	187	144	2.97	97.4
**45**	− 0.200	232	116	9.1	92.0

### Benzimidazoles as corrosion inhibitors for zinc

Shanbhag et al. found that benzimidazoles 2-mercaptobenzimidazole **11**, 2-hydroxy-benzimidazole **40**, 2-hydroxy-5-nitro-benzimidazole **46** and ethyl 2-(benzimidazolyl-2-thio) acetate **47** reduced the rate of corrosion of zinc considerably in 0.1 M HCl [47] by electrochemical method. The maximum inhibition efficiency of 93% was observed in the presence of **47** at 0.5 mM. Scanning electron microscopy (SEM) images reveal the existence of organic film on metal surface in presence of inhibitors.

### Quantum chemical assessment of benzimidazoles as corrosion inhibitors

A number of articles only refer to the theoretical prediction of the use of benzimidazoles as CIs [48–52]. Quantum chemical calculations, density functional theory (DFT) molecular dynamics simulations and QSAR analyses, were used to research the abilities for being a corrosion inhibitor of a benzimidazole. Roque et al. show that applying the DFT theory reveals the formation of an adsorption layer over the metallic surface, due passing of the π density from delocalization region (N_1_=C_2_=N_3_) through its HOMO orbital to the metal LUMO orbital [50]. Density functional theory (DFT) was successfully applied to describe the structural importance of corrosion inhibitor and its adsorption efficiency on metal surfaces [52].

### Polar functional groups grafted on the benzimidazole nucleus

The efficiency of the benzimidazole inhibitors strongly depends on their ability to form complexes with the metal because they act by adsorption on the metal surface. The polar functional groups containing sulfur, oxygen and nitrogen and the π electrons serve as the chelating centre for chemical adsorption. The inhibition efficiency of organic compounds containing different donor atoms is in the sequence S > N > O [53]. Several quantum-chemistry studies have been performed that relate inhibition efficiency to the molecular properties of benzimidazoles. Obayes et al. demonstrated that presence of nitro group (NO_2_) on corrosion inhibitor molecules decreases the IE (79.21% for 5-nitro-benzimidazole-2-thiol), while presence of the amino group (NH_2_) led to an increase in IE (101.38 for 5-amino-benzimidazole-2-thiol) [48]. Li et al. indicated that benzimidazole molecules with a dimethyl amino (N(CH_3_)_2_) group in positions “4” or “5” and a hydrazine (NH–NH_2_) group in position “2” are promising corrosion inhibitors [52]. Rodriguez-Clemente et al. show that the inhibition capacities of 1-(2-pyridinyl)-2-(*o*, *m*, *p*-hydroxyphenyl)benzimidazole indicate that presence of “OH” group at the *ortho* position, confers the best inhibition properties [38].

### Quantum chemical indices

The effects of the molecular structure on chemical reactivity have been studied extensively [53–57]. Quantum chemical indices calculated to predict that a compound is fitted to be a corrosion inhibitor are: energy of the highest occupied molecular orbital (E_HOMO_), energy of the lowest unoccupied molecular orbital (E_LUMO_), dipole moment (μ), polarizability (α), total natural charges (Q_total_), total Mulliken charges (Z_total_), adiabatic ionization potential (I_a_), adiabatic electron affinity (A_a_), Molar volume (V_i_), aromaticity-magnetism based on Nuclear Independent Chemical Shift (NICS), the number of electrons transferred (ΔN) and electrophilicity index (ω_a_) [52, 58–71]. The radial distribution function (RDF) study confirmed that both the neutral and protonated form of the inhibitor show significant interaction with the steel surface. Various interaction patterns are proposed to explain the corrosion efficiency of several benzimidazoles using the quantum chemical parameters mentioned above [16, 19, 20, 33, 43, 46].

### Aromaticity-magnetism based on Nuclear Independent Chemical Shift (Λ_NICS_)

The inhibitory activity depends on the aromaticity of the benzimidazole systems. Thus, the delocalization of the π-electrons in the aromatic systems plays a central role in the adsorption of the compounds on the metal surface. The delocalized π-electrons are transferred to the vacant orbitals of the metal atoms, establishing stable coordination bonds [20, 21, 32, 52].

### Frontier orbitals (HOMO, LUMO) and inhibition efficiency

The energy of HOMO (E_HOMO_) is associated with the electronic donating ability of a molecule. Therefore, an increase in the values of E_HOMO_ facilitates the adsorption, thus the inhibition efficiency, and indicates the availability of the molecule to donate electrons to an appropriate acceptor with empty molecular orbitals. The energy of LUMO (E_LUMO_) indicates the ability of the molecule to accept electrons. The parameter that determines chemical reactivity and kinetic stability of the molecule is the frontier orbital gap. A small frontier orbital gap defines a molecule with high chemical reactivity, low kinetic stability and more polarisable [72, 73]. The lower the value of E_LUMO_, the more probable it is that the molecule accepts electrons. A molecule with low energy gap ΔE = E_LUMO_ − E_HOMO_ will possess good inhibition efficiency, because the energy to remove an electron from the last occupied orbital will be low [53]. These correlations are made also considering the terms of ionization potential (I_a_)—the ability of a species to donate electrons; and the electronic affinity (A)—the ability of a species to accept electrons.

### The number of electrons transferred (ΔN)

The number of electrons transferred (ΔN) from the inhibitor molecule (donor) to the metallic atom (acceptor) indicates the tendency of a molecule to donate electrons. A higher value of ΔN indicates the greater tendency of a molecule to donate electrons [52]. According to Dibetsoe et al. negative values of ΔN_a_ suggest that the inhibition of metal corrosion is predominantly controlled by retro-donation from the metal to the inhibitor molecule [68]. According to Lukovits et al. [69] and to other recent studies [64, 70], a value of ΔN_a_ less than 3.6 indicates that the inhibition efficiencies of an inhibitor tend to increase by increasing the electron-donating ability to the metal surface.

### Dipole moment (μ) and inhibition efficiency

Several authors stated that inhibition efficiency increases with increasing values of the dipole moments, by influencing the transport process through the adsorbed layer [20, 53]. However, Zhang et al. reported that the low polarity species, such as **29**, are more efficient corrosion inhibitors than higher polarity species, like the protonated form of **29**, since the last is surrounded by a denser water layer compared to **29** [31].

### Total charge (Q_total_) or Mulliken population analysis (Z_total_)

The total negative charge represents the amount of charge carried by all non-hydrogen atoms of the benzimidazole. A larger total charge indicates a greater tendency for the molecule to be adsorbed on a metal surface [12, 52]. Therefore, the efficiency of an organic inhibitor depends of the many factors, such as chemical structure; size of the organic molecule; aromaticity and/or conjugated bonding; type and number of bonding atoms or groups in the molecule (either π or σ); nature and the charges of the metal surface of adsorption, such as mode like bonding strength to metal substrate; ability for a layer to become compact or cross-linked; capability to form a complex with the atom as a solid in the metal lattice; type of the electrolyte solution like adequate solubility in the environment [58–63, 74–76]. El-Hajjaji selected 1-octyl-2-(octylthio)-1*H*-benzimidazole because it can readily adsorb onto the steel surface, due to a positively charged nitrogen atom; also, the long hydrocarbon chain is likely to form a hydrophobic network which can further reduce the interaction between the metal and the corrosive environment [35].

Shi et al. found that the Mulliken atomic charges indicate that the adsorption of CIs occurs mostly through benzene ring and the lone pair electrons of the “nitro” atoms [77]. Similar results were reported by Baskar group for a series of benzimidazoles [78]. Sun et al. show two types of adsorption by calculating charges on the atoms: parallel adsorption, when the molecule is chemosorbed on the metal surface and perpendicular adsorption, in the case of dehydrogenated molecules chemosorbed [79]. The inhibition efficiencies are explained in theoretical terms by chain length, relative effects of amido and sulphonamido groups, spatial orientations, structural factors, energy gap between the frontier orbitals and charges on atoms for a series of benzimidazoles [26, 80].

### Substitution in the “2” position at benzimidazoles

Substitution in the “2” position at benzimidazoles, respectively 2-substituted benzimidazoles, have a better inhibitory activity than the unsubstituted benzimidazoles at position “2”, as can be seen from several studies [1, 16, 35], both experimental [16, 23, 27, 38, 40, 49] and theoretical [19, 26, 50, 72]. The superior inhibitory activity of the 2-substituted benzimidazoles compared to the unsubstituted benzimidazoles in position “2” can be concluded from the comparative inhibitory efficiency data mentioned in Table 2. Thus, if for benzimidazole **1**, not substituted in the “2” position, the maximum inhibitory efficiency is 60%, for 2-Aminobenzimidazole **10** and 2-Mercaptobenzimidazole **11**, the maximum inhibitory efficiency is much higher, of 86.8% and respectively 97.0% [22].

### Mechanisms of corrosion inhibition for benzimidazole compounds

**Fig. 22:**
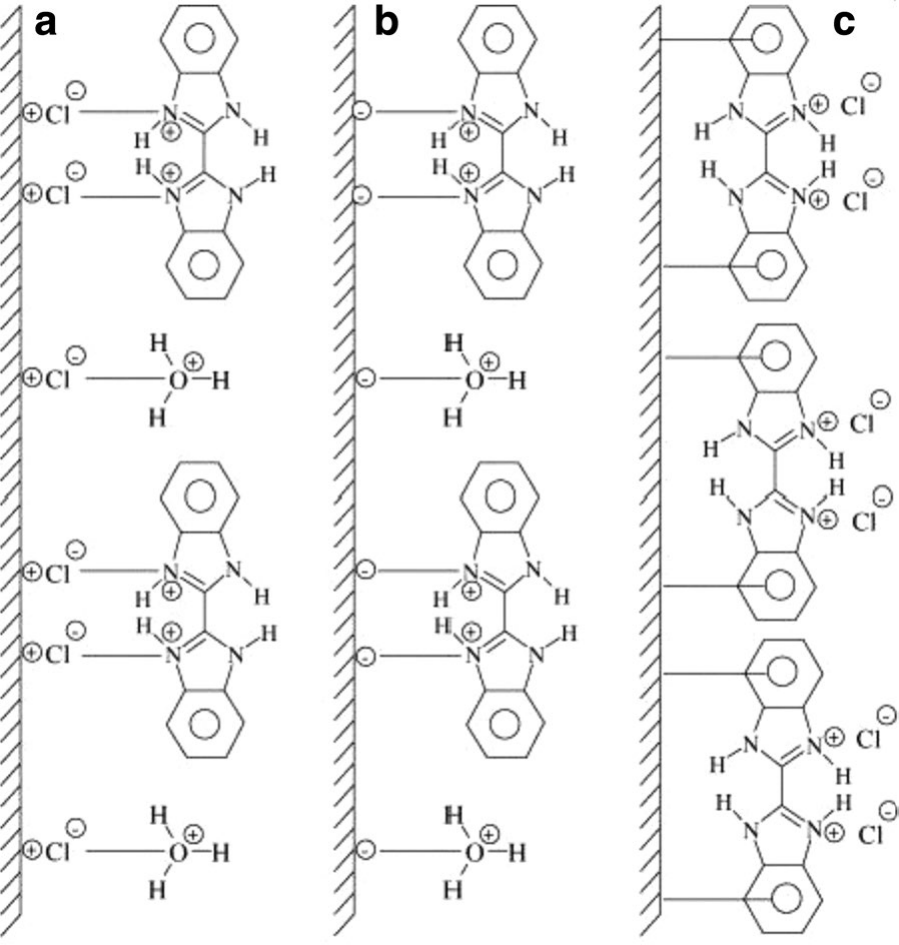
Schematic representation of adsorption behaviour of bis(benzimidazole) on mild steel in 1 M HCl solution: **a** mild steel surface with positive charge, **b** mild steel surface with negative charge and **c** mild steel surface at potential of zero charge (Reproduced with permission from Elsevier, Ref. [81])

**Fig. 23:**
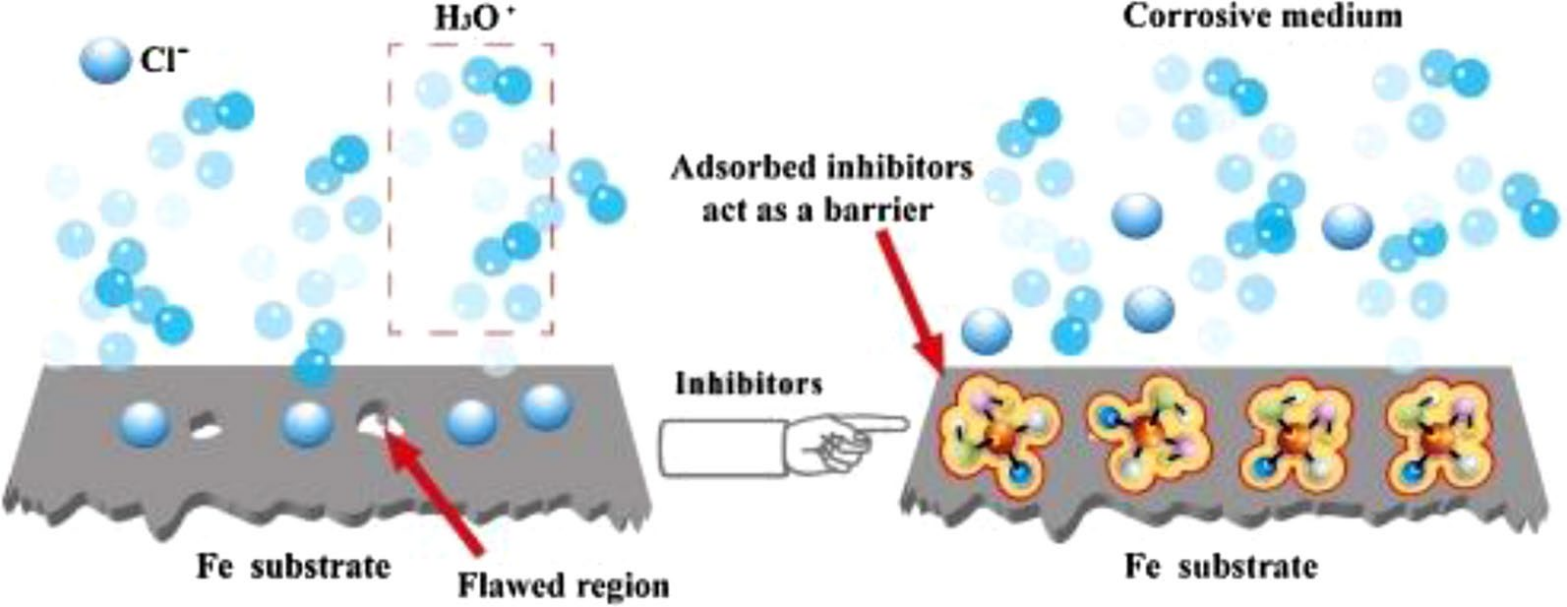
Schematic diagram of the corrosion inhibition mechanismin acidic media (reproduced with permission from Elsevier, Ref. [80])

There are many mechanisms proposed for the inhibition of metal corrosion by benzimidazole inhibitors. The first stage in the action mechanism of the inhibitor in the aggressive acid media is its adsorption on the metal surface [81]. The parameters implied in the adsorption of the organic inhibitors on the metal surface are: (1) the structure of the inhibitor which confers specific charges on the atoms and some type of interaction between metallic surface and organic substance; (2) the corrosive electrolyte (acid or basic); (3) nature of metal surface [82, 83]. The formation of donor–acceptor surface complexes between π-electrons of an inhibitor and the vacant d-orbital of metal was formulated in many inhibition studies [80, 84, 85]. Thus, the adsorption of the bis(benzimidazole) as neutral molecules on the metal surface can occur by displacement of water molecules from the metal surface and sharing of electrons between the nitrogen atoms and the metal surface. A schematic representation of the adsorption behaviour of bis(benzimidazole) on mild steel in 1 M HCl solution is shown in Fig. 22. Three kinds of species can be adsorbed on a mild steel surface when is immersed in dilute solution of HCl containing bis(benzimidazole): (a) the chloride ions will first be adsorbed on the metal surface, if the metal surface is positively charged and a close packed triple layer will form on the metal surface which inhibit the entry of iron ions to the solution (Fig. 22a); (b) the benzimidazole compound is directly adsorbed on the metal surface if the metal surface is negatively charged (Fig. 22b); (c) when the metal surface has the potential surface charge zero, none of the ions (neither cations nor anions) adsorb on the surface through their ionic centre (Fig. 22c). Another proposed scheme of the inhibition mechanism in acidic is shown in Fig. 23. The inhibitor molecules move quickly to the damaged regions to form strong coordination bonds with Fe atoms, by adding a certain concentration of corrosion inhibitors to the corrosive media. Consequently a protective barrier is formed to prevent the attack corrosive particles (such as Cl^−^, H_3_O) on Fe surface.

## Conclusion

In this paper we presented the syntheses of the tested benzimidazoles as corrosion inhibitors, the experimental conditions, the proposals of mechanisms of interaction between the inhibitor and the protected surface, as well as the theoretical approaches of these molecules. It has been shown that benzimidazoles are good and very good corrosion inhibitors for extremely aggressive, corrosive acidic media such as 1 M HCl, 1 M HNO_3_, 1.5 M H_2_SO_4_, 30% acetic acid, basic, as 0.1 M NaOH or solutions of salts such as 3% NaCl and NACE brine ID196. Also the metals tested are diverse, from the most searching, steels, mild steel and carbon steel, pure metals like Fe, Al, Cu, Zn, and alloys. EIS measurements suggest the adsorptive property of the benzimidazoles, that form a physical barrier on the metal surface hindering charge transfer reactions at the metal-solution interface. Generally, all these benzimidazole derivatives act as mixed type inhibitors, exhibit stronger inhibitive effect on the cathodic reaction than on the anodic one. SEM and AFM images confirm the adsorption of the inhibitors on the metal surface, therefore the protection of metals. Benzimidazoles can have very good inhibition efficiencies of up to 97–99% for concentrated acidic solutions of hydrochloric acid or sulfuric acid. Experimental methods for investigating corrosion processes and benzimidazole inhibitors have been presented. Polar groups, quantum parameters like, the energy of the HOMO and LUMO orbitals, aromaticity-magnetism based on Nuclear Independent Chemical Shift, dipole moment and total charge, are important parameters that define the anti-corrosion capacity of an inhibitor. We hope this study will open new horizons in the science of organic corrosion inhibitors to be applied even at industrial level.

## Data Availability

The datasets used and/or analyzed during the current study are available from the corresponding author on reasonable request.

## Abbreviations

CIs: corrosion inhibitors
PDA: 1,2-diaminobenzene
WL: weight loss
EIS: electrochemical impedance spectroscopy
LSW: linear sweep voltammetry
SEM: scanning electron microscopy
AFM: atomic force microscopy
IE: inhibition efficiency
HOMO: high occupied molecular orbital
LUMO: low unoccupied molecular orbital
PP: potentiodynamic polarization
CS: carbon steel
MS: mild steel
FTIR: Fourrier Transform infrared
UV–VIS: ultraviolet–visible
DFT: density functional theory
RDF: radial distribution function

## References

[1] Finsgar M, Jackson J (2014). Application of corrosion inhibitors for steels in acidic media for the oil and gas industry: a review. Corros Sci.

[2] Guttierez E, Rodriguez JA, Cruz-Borboll J, Alvarado-Rodriguez JG, Thangarasu P (2016). Development of a predictive model for corrosion inhibition of carbon steel by imidazole and benzimidazole derivatives. Corros Sci.

[3] Hamadi L, Mansouri S, Oulmi K, Kareche A (2018). The use of amino acids as corrosion inhibitors for metals: a review. Egypt J Petrol.

[4] Chen Y, Xing W, Wang L, Chen L (2019). Experimental and electrochemical research of an efficient corrosion and scale Inhibitor. Materials.

[5] El Ibrahimi B, Jmiai A, Bazzi L, El Issami S (2017). Amino acids and their derivatives as corrosion inhibitors for metals and alloys. Arab J Chem.

[6] Bansal Y, Silakari O (2012). The therapeutic journey of benzimidazoles: a review. Eur J Med Chem.

[7] Akhtar W, Khan MF, Verma G, Shaquiquzzaman M, Rizvi MA, Mehdi SH, Akhter M, Alam MM (2017). Therapeutic evolution of benzimidazole derivatives in the last quinquennial period. Eur J Med Chem.

[8] Horak E, Kassal P, Steinberg M (2017). Benzimidazole as a structural unit in fluorescent chemical sensors: the hidden properties of a multifunctional heterocyclic scaffold. Supramol Chem.

[9] Tayade RP, Sekar N (2016). Benzimidazole-thiazole based NLOphoric styryl dyes with solid state emission—synthesis, photophysical, hyperpolarizability and TD-DFT studies. Dyes Pigm.

[10] Emler S, Scholze M, Kortenkamp A (2013). Seven benzimidazole pesticides combined at sub-threshold levels induce micronuclei in vitro. Mutagenesis.

[11] Yuan S, Guo X, Aili D, Pan C, Li Q, Fang J (2014). Poly(imidebenzimidazole)s for high temperature polymer electrolyte membrane fuel cells. J Membr Sci.

[12] Mousavi M, Safarizadeh H, Khosravan A (2012). A new cluster model based descriptor for structure-inhibition relationships: a study of the effects of benzimidazole, aniline and their derivatives on iron corrosion. Corros Sci.

[13] Vinutha MR, Venkatesha TV (2016). Review on mechanistic action of inhibitors on steel corrosion in acidic media. Portug Electrochim Acta.

[14] Wright JB (1951). The chemistry of the benzimidazoles. Chem Rev.

[15] Preston PN (1974). Synthesis, reactions, and spectroscopic properties of benzimidazoles. Chem Rev.

[16] Dutta A, Saha SK, Adhikari U, Banerjee P, Sukul D (2017). Effect of substitution on corrosion inhibition properties of 2-(substituted-phenyl) benzimidazole derivatives on mild steel in 1 M HCl solution: a combined experimental and theoretical approach. Corros Sci.

[17] Yadav M, Kumar S, Purkait T, Olasunkanmi LO, Bahadur I, Ebenso EE (2016). Electrochemical, thermodynamic and quantum chemical studies of synthesized benzimidazole derivatives as corrosion inhibitors for N80 steel in hydrochloric acid. J Mol Liq.

[18] Yildirim A, Ozturk S, Cetin M (2013). Long-chain alkylthia-benzimidazoles as corrosion Inhibitors for carbon steel in H_2_SO_4_ solution. Phos Sulf Silicon Relat Elem.

[19] Singh A, Ansari KR, Quraishi MA, Lgaz H (2018). Effect of electron donating functional groups on corrosion inhibition of J55 steel in a sweet corrosive environment: experimental, density functional theory, and molecular dynamic simulation. Materials.

[20] Dutta A, Saha SK, Adhikari U, Banerjee P, Sukul D (2015). Correlating electronic structure with corrosion inhibition potentiality of some bis-benzimidazole derivatives for mild steel in hydrochloric acid: combined experimental and theoretical studies. Corros Sci.

[21] Ibrahim MM, Amin MA, Ichikawa K (2011). Synthesis and characterization of benzimidazole-based zinc complexes as structural carbonic anhydrase models and their applications towards CO_2_ hydration. J Mol Struct.

[22] Popova A, Sokolova E, Raicheva S, Christov M (2003). AC and DC study of the temperature effect on mild steel corrosion in acid media in the presence of benzimidazole derivatives. Corros Sci.

[23] Mahdavian M, Ashhari (2010). Corrosion inhibition performance of 2-mercaptobenzimidazole and 2-mercaptobenzoxazole compounds for protection of mild steel in hydrochloric acid solution. Electrochim Acta.

[24] Wang X, Zang H, Wang F (2011). An investigation of benzimidazole derivative as corrosion inhibitor for mild steel in different concentration HCl solutions. Corros Sci.

[25] Wang X, Wan Y, Zeng Y, Gu Y (2012). Investigation of benzimidazole compound as a novel corrosion inhibitor for Mild Steel in hydrochloric acid solution. Int J Electrochem Sci.

[26] Dutta A, Panja SS, Nandi MM, Sukul D (2015). Effect of optimized structure and electronic properties of some benzimidazole derivatives on corrosion inhibition of mild steel in hydrochloric acid medium: electrochemical and theoretical studies. J Chem Sci.

[27] Zhang F, Tang Y, Cao Z, Jing W, Wu Z, Chen Y (2012). Performance and theoretical study on corrosion inhibition of 2-(4-pyridyl)-benzimidazole for mild steel in hydrochloric acid. Corros Sci.

[28] Thang Y, Zhang F, Hu S, Cao Z, Wu Z, Jing W (2013). Novel benzimidazole derivatives as corrosion inhibitors of mild steel in the acidic media. Part I: gravimetric, electrochemical, SEM and XPS studies. Corros Sci.

[29] Ramya K, Mohan R, Joseph A (2014). Interaction of benzimidazoles and benzotriazole: its corrosion protection properties on Mild Steel in hydrochloric acid. JMEPEG.

[30] Lgaz H, Salghi R, Jodeh S (2016). Corrosion inhibition potentiality of some benzimidazole derivatives for mild steel in hydrochloric acid: electrochemical and weight loss studies. Int J Corros Scale Inhib.

[31] Zhang D, Tang Y, Qi S, Dong D, Cang H, Lu G (2016). The inhibition performance of long-chain alkyl-substituted benzimidazole derivatives for corrosion of mild steel in HCl. Corros Sci.

[32] Zheng X, Zhang S, Li W, Yin L, He J, Wu J (2014). Investigation of 1-butyl-3-methyl -1*H*-benzimidazolium iodide as inhibitor for mild steel in sulfuric acid solution. Corros Sci.

[33] Aljourani J, Golozar MA, Raeis K (2010). The inhibition of carbon steel corrosion in hydrochloric and sulfuric acid media using some benzimidazole derivatives. Mater Chem Phys.

[34] Garcia-Ochoa E, Guzman-Jimenez SJ, Guadalupe Hernandez J, Pandiyan T, Vasquez-Perez JM, Cruz-Borbolla J (2016). Benzimidazole ligands in the corrosion inhibition for carbon steel in acid medium: dFT study of its interaction on Fe_30_ surface. J Mol Struct.

[35] El-Hajjaji F, Merimi I, El Ouasif L, El Ghoul M, Achour R, Hammouti B, Belghiti ME, Chauchan DS, Quraishi MA (2019). 1-Octyl-2-(octylthio)-1*H*-benzimidazole as a new and effective corrosion Inhibitor for carbon steel in 1 M HCl, Portugal. Electrochim Acta.

[36] Solmaz R (2010). Investigation of the inhibition effect of 5-((E)-4-phenylbuta-1,3- dienylideneamino) -1,3,4-thiadiazole-2-thiol Schiff base on mild steel corrosion in hydrochloric acid. Corros Sci.

[37] Behpour M, Ghoreishi SM, Soltani N, Salavati-Niasari M (2009). The inhibitive effect of some bis-N, S-bidentate Schiff bases on corrosion behaviour of 304 stainless steel in hydrochloric acid solution. Corros Sci.

[38] Rodriguez-Clemente E, Barrera-Pascual V, Cervantes-Huevas H, Aldana-Gonzales J, Uruchurtu-Chavarin J, Romero-Romo M, Palomar-Pardavé M (2018). New 1-(2-pyridinyl)-2-(*o*-, *m*-, *p*-hydroxyphenyl) benzimidazoles as corrosion inhibitors for API 5L X52 steel in acid media. Anti-Corros Methods Mater.

[39] Moreira RR, Soares TF, Ribeiro J (2014). Electrochemical Investigation of corrosion on AISI 316 Stainless Steel and AISI 1010 Carbon Steel: study of the behaviour of imidazole and benzimidazole as corrosion inhibitors. Adv Chem Eng Sci.

[40] Onyeachu IB, Obot IB, Sorour AA, Abdul-Rashid MI (2019). Green corrosion inhibitor for oilfield application I: electrochemical assessment of 2-(2-pyridyl) benzimidazole for API X60 steel under sweet environment in NACE brine ID196. Corros Sci.

[41] Khaled KF (2003). The inhibition of benzimidazole derivatives on corrosion of iron in 1 M HCl solutions. Electrochim Acta.

[42] Bereket G, Binarbasi A, Ogretir C (2004). Benzimidazole-2-tione and benzoxazole-2-tione derivatives as corrosion inhibitors for aluminium in hydrochloric acid. Anti-Corros Methods Mater.

[43] Chacko M, Nayak J (2015). Benzimidazole as corrosion inhibitor for heat treated 6061 Al-SiC_p_ composite in acetic acid. J Phys Conf Ser.

[44] Zhang D, Gao LX, Zhou GD (2004). Inhibition of copper corrosion in aerated hydrochloric acid solution by heterocyclic compounds containing a mercapto group. Corros Sci.

[45] Niamien PM, Kouasi HA, Trokoueri A, Essy FK, Sissouma D, Bokra Y (2012) Copper corrosion inhibition in 1 M HNO_3_ by two benzimidazole derivatives. Int Schol Res Net ID 623754

[46] Ozbay S, Yanardag T, Dincer S, Aksut AA (2014). Benzimidazole Schiff bases as corrosion inhibitors for copper and brass. Eur Int J Sci Technol.

[47] Shanbag AV, Venkatesha TV, Praveen M (2013). Benzimidazole derivatives as corrosion inhibitors for Zinc in acid solution. Prot Met Phys Chem.

[48] Obayes HR, Alwan GH, Alobaidy AHMJ, Al-Amiery AA, Kadhum AAH, Mohamad AB (2014). Quantum chemical assessment of benzimidazole derivatives as corrosion inhibitors. Chem Cent J.

[49] Xu B, Gong W, Zhang K, Yang W, Liu Y, Yin X, Ghi H, Chen Y (2015). Theoretical prediction and experimental study of 1-Butyl-2-(4-methylphenyl)benzimidazole as a novel corrosion inhibitor for mild steel inhydrochloric acid. J Taiwan Inst Chem E.

[50] Roque JM, Pandiyan T, Cruz J, Garcia-Ochoa E (2008). DFT and electrochemical studies of tris(benzimidazole-2-ylmethyl)amine as an efficient corrosion inhibitor for carbon steel surface. Corros Sci.

[51] Zhang J, Yu W, Yu L, Yan Y, Qiao G, Hu S, Ti Y (2011). Molecular dynamics simulation of corrosive particle diffusion in benzimidazole inhibitor films. Corros Sci.

[52] Li L, Zhang X, Gong S, Zhao H, Bai Y, Li Q, Ji L (2015). The discussion of descriptors for the QSAR model and molecular dynamics simulation of benzimidazole derivatives as corrosion inhibitors. Corros Sci.

[53] Obot IB, Obi-Egbedi NO (2010). Theoretical study of benzimidazole and its derivatives and their potential activity as corrosion inhibitors. Corros Sci.

[54] Khalil N (2003). Quantum Chemical Approach of Corrosion Inhibition. Electrochim Acta.

[55] Lukovits I, Palfi K, Bako I, Kalman E (1997). LKP model of the inhibition mechanism of thiourea compounds. Corros Sci.

[56] Bentiss F, Traisnel M, Vezin H, Lagrenee M (2003). Linear resistance model of the inhibition mechanism of steel in HCl by triazole and oxadiazole derivatives: structure–activity correlations. Corros Sci.

[57] Abdul-Ahad PG, Al-Madfai SHF (1989). Elucidation of corrosion inhibition mechanism by means of calculated electronic indexes. Corrosion.

[58] Marinescu M, Emandi A, Marton G, Cinteza LO, Constantinescu C (2015). Structural studies and optical nonlinear response of some pyrazole-5-ones. Nanosci Nanotechnol Lett.

[59] Marinescu M, Tudorache DG, Marton G, Zalaru CM, Popa M, Chifiriuc MC, Stavarache C, Constantinescu C (2017). Density functional theory molecular modeling, chemical synthesis, and antimicrobial behaviour of selected benzimidazole derivatives. J Mol Struct.

[60] Marinescu M, Cinteza LO, Marton G, Măruţescu L, Chifiriuc MC, Constantinescu C (2017). Density functional theory molecular modeling and antimicrobial behaviour of selected 1,2,3,4,5,6,7,8-octahydroacridine-N(10)-oxides. J Mol Struct.

[61] Xu S, Zhang S, Guo L, Feng L, Tan B (2019). Experimental and theoretical studies on the corrosion inhibition of carbon steel by two indazole derivatives in HCl medium. Materials.

[62] Al-Azawi KF, Al-Baghdadi SB, Mohamed AZ, Al-Amiery AA, Abed TK, Mohammed SA, Kadhum AAH, Mohamad AB (2016). Synthesis, inhibition effects and quantum chemical studies of a novel coumarin derivative on the corrosion of mild steel in a hydrochloric acid solution. Chem Cent J.

[63] Feng L, Zhang S, Qiang Y, Xu Y, Guo L, Madkour LH, Chen S (2018). Experimental and theoretical investigation of Thiazolyl Blue as a corrosion inhibitor for copper in neutral sodium chloride solution. Materials.

[64] Asegbeloyin JN, Ejikeme PM, Olasunkanmi LO, Adekunle AS, Ebenso EE (2015). A novel Schiff base of 3-acetyl-4-hydroxy-6-methyl-(2*H*)pyran-2-one and 2,2′-(ethylenedioxy) diethylamine as potential corrosion inhibitor for Mild Steel in acidic medium. Materials.

[65] de Amorim Lima NM, Avila HJC, Marchiori CFN, Sampaio SG, Mota JPF, Ribeiro VGP, da Silva Clemente C, Mele G, Cremona M, Mazzetto SE (2019). Light-emitting porphyrin derivative obtained from a subproduct of the cashew nut shell liquid: a promising material for OLED applications. Materials.

[66] Sun L, Shu S, Zhou Y, Hou S, Liu Y, Ke Z (2018). Regulating the optoelectronic properties of nickel dithiolene by the substituents: a theoretical study. Materials.

[67] Nwankwo HU, Ateba CN, Olasunkanmi LO, Adekunle AS, Isabirye DA, Onwudiwe DC, Ebenso EE (2016). Synthesis, characterization, antimicrobial studies and corrosion inhibition potential of 1,8-dimethyl-1,3,6,8,10,13-hexaazacyclotetradecane: experimental and quantum chemical studies. Materials.

[68] Dibetsoe M, Olasunkanmi LO, Fayemi OE, Yesudass S, Ramaganthan B, Bahadur I, Adekunle AS, Kabanda MM, Ebenso EE (2015). Some phthalocyanine and naphthalocyanine derivatives as corrosion inhibitors for aluminium in acidic medium: experimental, quantum chemical calculations, QSAR studies and synergistic effect of iodide ions. Molecules.

[69] Lukovits I, Kálmán E, Zucchi F (2001). Corrosion inhibitors-correlation between electronic structure and efficiency. Corrosion.

[70] Olasunkanmi LO, Obot IB, Kabanda MM, Ebenso EE (2015). Some quinoxalin-6-yl derivatives as corrosion inhibitors for mild steel in hydrochloric acid: experimental and theoretical studies. J Phys Chem C.

[71] Almansour AI, Arumugam N, Kumar RS, Soliman SM, Altaf M, Ghabbour HA (2016). Synthesis, spectroscopic, X-ray diffraction and DFT studies of novel benzimidazole fused-1,4-oxazepines. Molecules.

[72] Ghani NTA, Mansour AM (2012). Molecular structures of 2-arylaminomethyl-1*H*-benzimidazole: spectral, electrochemical, DFT and biological studies. Spectrochim Acta A.

[73] Lavanya K, Saranya J, Chitra S (2018). Recent reviews on quinoline derivative as corrosion inhibitors. Corros Rev.

[74] Dariva CG, Aliofkhazraei M (2014). Galio AF Corrosion inhibitors—principles, mechanisms and applications. Developments in corrosion protection.

[75] Rivetti MLS, Netto JSA, Amorim Junior MS, Ribeiro DV, Aliofkhazraei M (2018). Corrosion Inhibitors for Reinforced Concrete. Corrosion inhibitors, principles and recent application.

[76] Tuzun B (2019). Investigation of benzimidazole derivates as crrosion inhibitor by DFT. Cumhuriyet Sci J.

[77] Shi H, Xu B, Zhu HJ (2016). Electrochemical and theoretical studies of 1-alkyl-2-substituted benzimidazoles as corrosion inhibitors for Carbon Steel surface in HCl medium. Chin J Struct Chem.

[78] Baskar R, Lgaz H, Salghi R (2019). Heterocyclic compounds as corrosion inhibitors for mild steel: a review. Chem Sci Eng Res.

[79] Sun S, Geng Y, Tian L, Chen S, Yan Y, Hu S (2012). Density functional theory study of imidazole, benzimidazole and 2-mercaptobenzimidazole adsorption onto clean Cu(111) surface. Corros Sci.

[80] Guo L, Obot IB, Zheng X, Shen X, Qiang Y, Kaya S, Kaya C (2017). Theoretical insight into an empirical rule about organic corrosion inhibitors containing nitrogen, oxygen, and sulfur atoms. Appl Surf Sci.

[81] Abboud Y, Abourriche A, Saffaj T, Berrada M, Charrouf M, Bennamara A, Cherqaoui A, Takky D (2006). The inhibition of mild steel corrosion in acidic medium by 2,2′-bis(benzimidazole). Appl Surf Sci.

[82] Wang F, Zhang Z, Wu S, Jiang J, Chu H (2019). Effect of inhibitor on adsorption behavior and mechanism of micro-zone corrosion on carbon steel. Materials.

[83] Taghavikish M, Dutta NK, Choudhury NR (2017). Emerging Corrosion Inhibitors for Interfacial Coating (Review). Coatings.

[84] Mohan P, Usha R, Paruthimal Kalaignan G, Muralidharan VS (2013). Inhibition effect of benzohydrazide derivatives on corrosion behaviour of mild steel in 1 M HCl. J Chem.

[85] Marinescu M, Marinescu M (2019). Introductory chapter: short insight in synthesis and applications of benzimidazole and its derivatives. Chemistry and applications of benzimidazole and its derivatives.

